# Supplementary material for: Diselenide crosslinks for enhanced and simplified oxidative protein folding
Source: Commun Chem. 2021 Mar 5;4:30. doi: 10.1038/s42004-021-00463-9 (PMC9814483; doi:10.1038/s42004-021-00463-9)
Supplement: Supplementary file 1 — Supplementary Information [file 42004_2021_463_MOESM1_ESM.pdf]

Diselenide Crosslinks for Enhanced and Simplified Oxidative Protein Folding

Reem Mousa, Taghreed Hidmi, Sergei Pomyalov, Shifra Lansky, Lareen Khouri, Deborah E. Shalev, Gil Shoham*, and Norman Metanis*

Supporting Information

Table of Contents

1. Materials and Methods 3
2. High Performance Liquid Chromatography (HPLC) 3
3. Mass Spectrometry (MS) and HR-MS 4
4. Experimental section 4
   4.1. Peptide synthesis 4
      Synthesis of Hir(39-65) 5
      Synthesis of Hir(39-65)(C39U) 6
      Synthesis of Hir(1-38)-COSR, Hir(1-38)(C6U/C14U)-COSR, Hir(1-38)(C16U/C28U)-COSR and Hir(1-38)(C22U)-COSR 7
      Non-native Hir(1-38)(C6U/C16U)-Nbz 12
   4.2. Native chemical ligations (NCL) 14
   4.3. Oxidative folding 22
   4.4. X-Ray crystallography 24
   Crystallization and crystallographic data collection 24
   The Hir(C16U/C28U)-Thrombin complex (Complex-1) 24
   The Hir(C6U/C14U)-Thrombin complex (Complex-2) 25
   The Hir(C22U/C39U)-Thrombin complex (Complex-3) 25
   Supplementary discussion-Further structural analysis 28
      Overall structures 28
      The diselenide bonds and their environments 29
      Intramolecular hydrogen bonds within the Se-Hir structures 31
      Intermolecular hydrogen bonds between the Se-Hir analogs and Thrombin 36
      RMS deviation by residue 39
      Implications on structural differences 40
   4.5. 2 D-NMR of WT-Hir and Hir(C6U/C14U) 42
   4.6. Inhibition assays 43
5. HR-MS results 45

Supplementary methods

1. Materials and Methods

Buffers for both ligation reactions and kinetic experiments were prepared using MilliQ water (Millipore, Merck). Ultrapure guanidinium chloride (Gn·HCl, MP Biomedicals, LLC, France) was used in all ligation buffers. $Na_2HPO_4 \cdot 12H_2O$, tris(2-carboxyethyl)phosphine hydrochloride (TCEP·HCl), 4-mercaptophenylacetic acid (MPAA), 2,2`-Dithiobis (5-nitropyridine) (DTNP), sodium ascorbate, *D,L*-dithiothreitol (DTT), triisopropylsilane (TIPS), acetylacetone (Acac), oxidized *L*-Glutathione (GSSG), N-Benzoyl-Phe-Val-Arg-*p*-nitroanalide hydrochloride, thrombin from bovine plasma were purchased from Merck (Jerusalem, Israel). All Fmoc-amino acids were obtained from CS Bio Co. (Menlo Park, CA) or Matrix innovation (Quebec City, Canada), with the following side chain protecting groups: Arg(Pbf), Asp(OtBu), Glu(OtBu), Ser(tBu), Thr(tBu), Cys(Trt), Lys(Boc), Tyr(tBu), Asn(Trt). (Pbf = 2,2,4,6,7- pentamethyl-2,3-dihydrobenzofuran-5-sulfonyl). TentaGel® R RAM resin (loading 0.18 mmol/g), Fmoc-Gln(Trt)-Wang resin (loading 0.19 mmol/g) and chlorotrityl resin (loading 1.8 mmol/g) were purchased from Rapp Polymer GmbH (Germany), GL Biochemical (China) or Chem-Impex (USA). *N,N,N',N'*-Tetramethyl-O-(6-chloro-1H-benzotriazol-1-yl)uronium hexafluorophosphate (HCTU) and Ethyl cyano(hydroxyimino)acetate (OxymaPure) were purchased from Luxembourg Biotechnologies Ltd. (Rehovot, Israel). All solvents: *N,N*-dimethylformamide (DMF), dichloromethane (DCM), acetonitrile (ACN), *N,N*-diisopropylethyl amine (DIEA), piperidine (Pip), diethyl ether (Et_2O) and trifluoroacetic acid (TFA) were purchased from Bio-Lab (Jerusalem, Israel) and were peptide synthesis, HPLC or ULC-grade. Fmoc-Sec(MoB)-OH was synthesised as reported previously.[1]

2. High Performance Liquid Chromatography (HPLC)

The analytical analyses were performed on a reverse-phase Waters Alliance HPLC with UV detector (220 nm and 280 nm) using an X-Bridge C4 column (300 Å, 3.5 µm, 4.6 ×150 mm) and an Atlantis T3 column (3 µm, 4.6 × 150 mm). Preparative and semi-preparative RP-HPLC was performed on a Waters LCQ150 system using XSelect C18 column (130 Å, 5 µm, 30 × 250 mm), X-Bridge BEH C4 (300 Å, 5 µm 19 × 150 mm) and X-Bridge BEH C4 (300 Å, 5 µm, 10 × 150 mm). Linear gradients of acetonitrile with 0.1% TFA (buffer B) and water with 0.1% TFA (buffer A) were used for all systems to elute

bound peptides. The flow rates were 1 mL/min (analytical), 3.34 mL/min (semi-preparative), 10 mL/min and 20 mL/min (C4 preparative and C18 preparative, respectively).

3. Mass Spectrometry (MS) and HR-MS

MS was performed on Thermo Scientific-LCQ Fleet Ion-Trap mass spectrometer. Peptide masses were calculated from the experimental mass to charge (m/z) ratios from the observed multiply charged species of a peptide using MagTran v1.03.

The HR-MS were recorded on a Q-ExactivePlus Orbitrap mass spectrometer (Thermo Scientific) with a ESI source and 140’000 FWHM, in a method with AGC target set to 1E6, and scan range was 400-2800 m/z. The raw data was deconvoluted by MagTran v1.03 software.

4. Experimental section

4.1. Peptide synthesis

General procedure for Fmoc-SPPS

Peptides were prepared manually or using an automated peptide synthesizer (CS136XT, CS Bio Inc. CA) typically on 0.25 mmol scale. Fmoc-amino acids (2 mmol) were activated with HCTU (2 mmol) and DIEA (4 mmol) for 5 min and coupled for 25 min, with constant shaking. Fmoc deprotection step was carried out with 20% piperidine in DMF for 2 x 5 min, and DMF was used for washing the resin. Fmoc-Sec(Mob)-OH was coupled manually using DIC/OxymaPure activation method.[1]

The sequence of Hirudin variant-1; UniProt - P01050

```
         10         20         30         40         50         60
VVYTDCTESG QNLCLCEGSN VCGQGNKCIL GSDGEKNQCV TGEGTPKPQS HNDGDFEEIP

EEYLQ
```

WT-Hirudin (WT-Hir) and its seleno-hirudin analogs were synthesised using two segments and one ligation reaction. The ligation site is in bold and underlined. The detailed syntheses of the segments and ligation reaction are described below.

Synthesis of Hir(39-65)

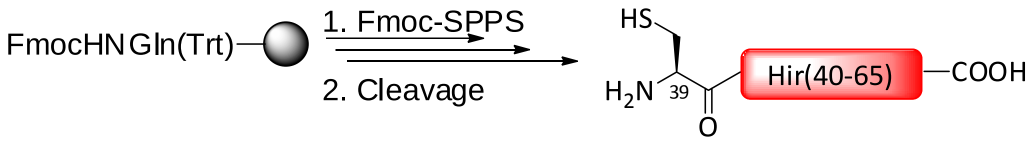

Scheme S1. Total chemical synthesis of Hir(39-65) by SPPS.

The synthesis of Hir(39-65) was carried out on Fmoc-Gln(Trt)-wang resin (0.19 mmol/g, 0.25 mmol scale) on an automated peptide synthesizer. Gly34 was manually coupled as Fmoc-(Dmb)Gly-OH (2.5 equiv Fmoc-(Dmb)Gly-OH,[2] 2.5 equiv HCTU and 5 equiv DIEA) and Asp33 was double coupled. Following the SPPS the peptide was cleaved and deprotected using TFA: TIPS: water (95: 2.5: 2.5). The peptide was purified using RP-HPLC (C18 column) to give pure fragment of Hir(39-65) in ~20 % yield (Scheme S1, Figure S1).

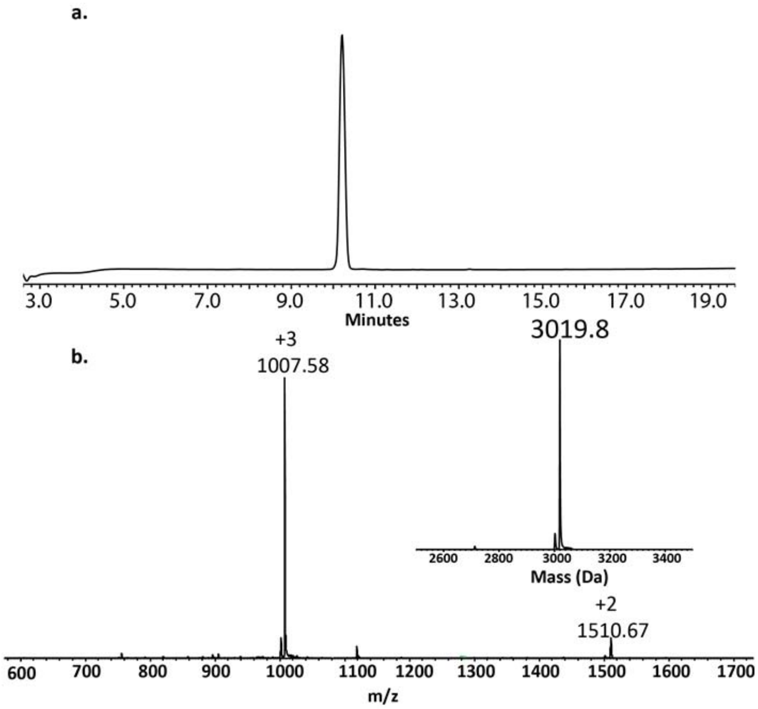

Figure S1. Characterization of the Hir(39-65) segment. **a**. HPLC analysis (220 nm); **b**. Corresponding ESI-MS, with its deconvoluted mass (inset) (obs. average 3019.8 ± 0.3 Da, calc. Da 3020.1)

Synthesis of Hir(39-65)(C39U)

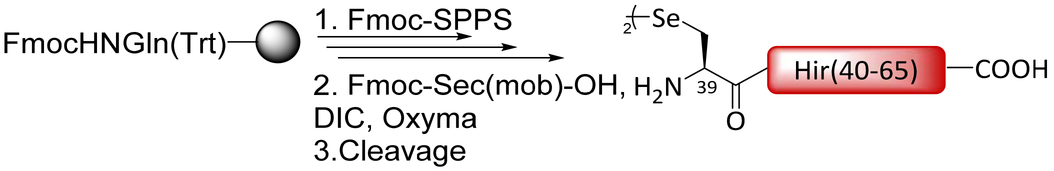

Scheme S2. Total chemical synthesis of Hir(39-65)(C39U) by SPPS

The synthesis of Hir(39-65)(C39U) was done in the same fashion as described for Hir(39-65). The Sec at position 39 was manually double coupled for 2 h (3.0 equiv Fmoc-Sec(Mob)-OH activated on ice for 5 min using 3.0 equiv OxymaPure and 2.9 equiv DIC) and treated with 20% of piperidine for Fmoc final deprotection[3]. The peptide was cleaved and purified to give Hir(39-65)(C39U) at ~13 % yield of pure peptide (Scheme S2, Figure S2). This analog was isolated as a dimer after HPLC purification, since it does not contain other Cys or Sec residues in its sequence. For analytical HPLC run, we treated a small amount of the peptide with TCEP and sodium ascorbate, which reduces the diselenide bonds, as is shown in Fig. S2.

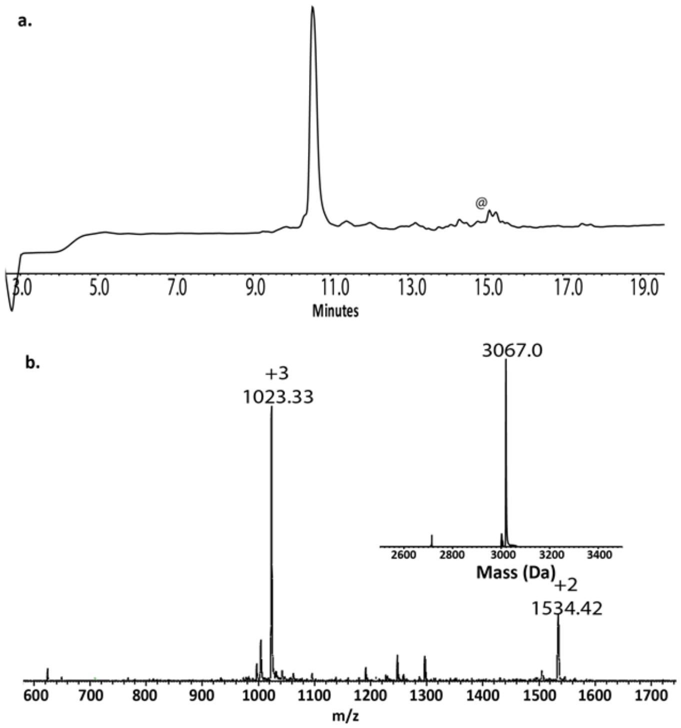

Figure S2. Characterization of the Hir(39-65)(C39U) segment. **a.** Analytical HPLC analysis (220 nm); **b**. The corresponding ESI-MS, with its deconvoluted mass (inset) (obs. average 3067.0 ± 0.1 Da, calc.

Da 3067.0). @ is corresponds to column impurities. For this run, the peptide was treated with TCEP and sodium ascorbate to keep it in the reduced form.

Synthesis of Hir(1-38)-COSR, Hir(1-38)(C6U/C14U)-COSR, Hir(1-38)(C16U/C28U)-COSR and Hir(1-38)(C22U)-COSR

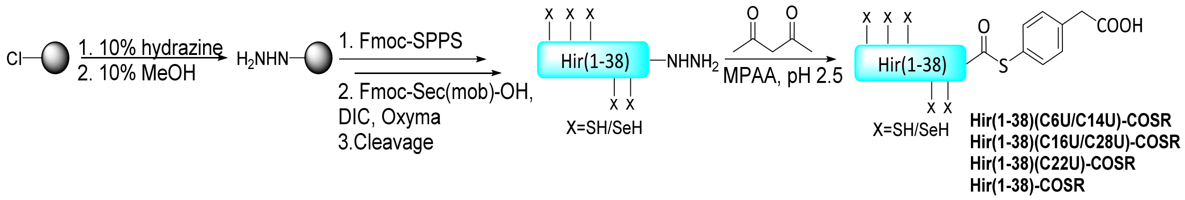

Scheme S3. Total chemical synthesis of Hir(1-38)-COSR, Hir(1-38)(C6U/C14U)-COSR, Hir(1-38)(C16U/C28U)-COSR and Hir(1-38)(C22U)-COSR by SPPS

The synthesis Hir(1-38)(C6U/C14U)-COSR, was carried out on Chlorotrityl resin, on a 0.25 mmol scale (1.8 mmol/g) was swelled in DMF for 1 h and treated twice with freshly prepared 10% hydrazine in DMF for 30 min and decanted.[4] The resin was washed well with DMF and then treated with 10% MeOH in DMF for 30 min. The hydrazide functionalized chlorotrityl resin was used for standard Fmoc-SPPS where the coupling of the amino acids held on an automated synthesizer. Gly34 was manually coupled as (Dmb)Gly (2.5 equiv Fmoc-(Dmb)Gly-OH,[2] 2.5 equiv HCTU and 5 equiv DIEA) and Asp33 was double coupled. Sec6 and Sec14 was manually coupled for 2 h (3.0 equiv Fmoc-Sec(Mob)-OH activated on ice for 5 min using 3.0 equiv OxymaPure and 2.9 equiv DIC). The cleavage was done in the presence of 2 equiv of DTNP,[5] using TFA:water:thioanisole (94:3:3) cocktail for 3-4 h.

<u>The conversion to thioester</u> was done by dissolving the peptide in PB buffer (200 mM, 6 M Gn·HCl, pH ~2.5) and treated with 50 equiv of acetylacetone (acac) and 200 equiv of MPAA for 4 h at room temperature.[6] Purification by RP-HPLC (C4 column) yielded ~4.3 % of pure peptide (Scheme S3, Figure S3).

Using the same manner, peptide Hir(1-38)(C16U/C28U)-COSR (Sec was substituted at position 16 and 28) and Hir(1-38)(C22U)-COSR (Sec substituted at position 22), were synthesised and purified to give ~6.6% and ~ 2% of pure peptide, respectively (Scheme S3, Figures S4 and S5).

The synthesis of Hir(1-38)-COSR followed the same procedure mentioned above (no Sec substitution) to yield 7.5% of pure peptide (Scheme S3, Figure S6).

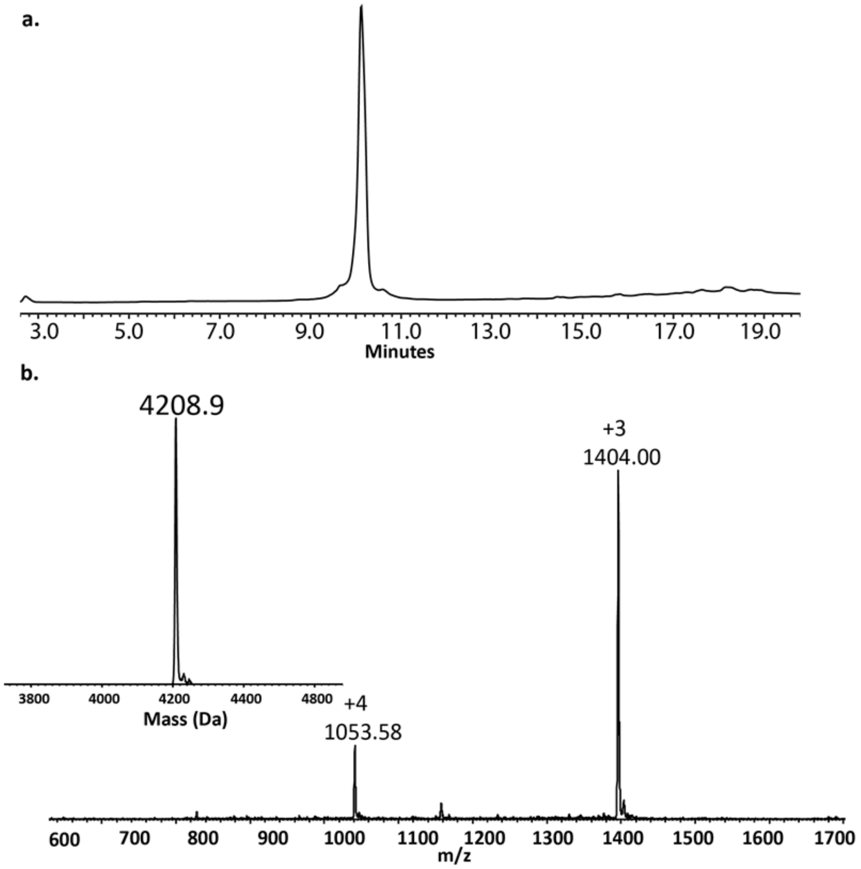

Figure S3. Characterization of the Hir(1-38)(C6U/C14U)-COSR segment. **a**. Analytical HPLC analysis of purified Hir(1-38)(C6U/C14U)-COSR (220 nm); **b**. The corresponding ESI-MS, with its deconvoluted mass (inset) (obs. average 4208.9 ± 0.5 Da, calc. 4209.3 Da). The peptide was purified with single diselenide crosslink and three reduced thiols.

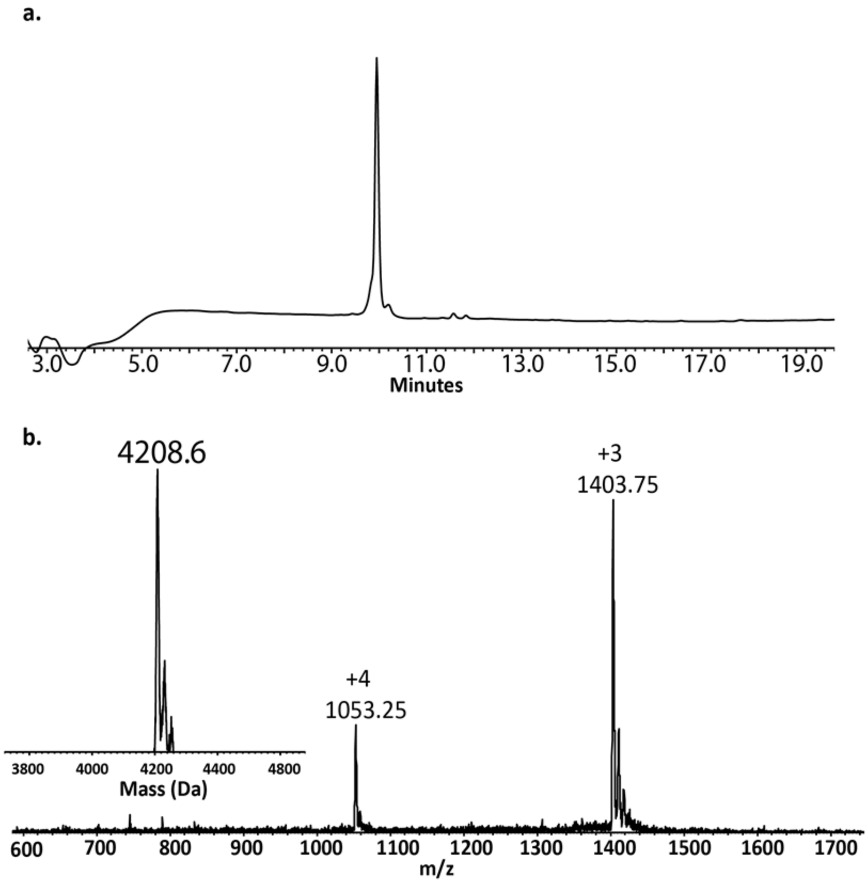

Figure S4. Characterization of the Hir(1-38)(C16U/C28U)-COSR segment **a.** Analytical HPLC analysis of purified Hir(1-38)(C16U/C28U)-COSR (220 nm); **b**. The corresponding ESI-MS, with its deconvoluted mass (inset) (obs. average 4208.6± 0.5 Da, calc. Da 4209.3). The peptide was purified with single diselenide crosslink and three reduced thiols.

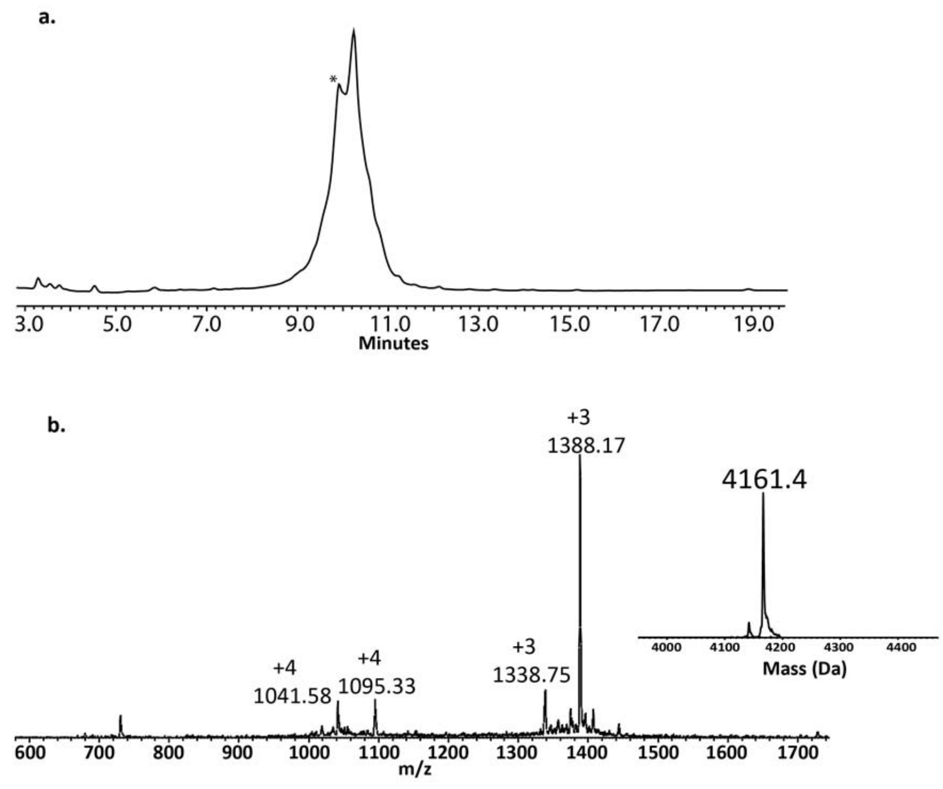

Figure S5. Characterization of the Hir(1-38)(C22U)-COSR segment. **a**. Analytical HPLC analysis of purified Hir(1-38)(C22U)-COSR (220 nm); **b**. and its corresponding ESI-MS, with its deconvoluted mass (inset) (obs. average 4161.4 ± 0.6 Da, calc. Da 4162.4). *This thioester suffered from hydrolysis side reaction; giving Hir(1-38)(C22U)-OH (-148.1 Da). The peptide was purified with one selenylsulfide crosslink and three reduced thiols, as such, a broad peak is observed due to the formation of a mixture selenylsulfide-containing isomers, in contrast to the other peptide analogs which gave a single isomer (sharp peak) containing a diselenide and three reduced thiols (see Fig. S3, S4 and S7 for comparison).

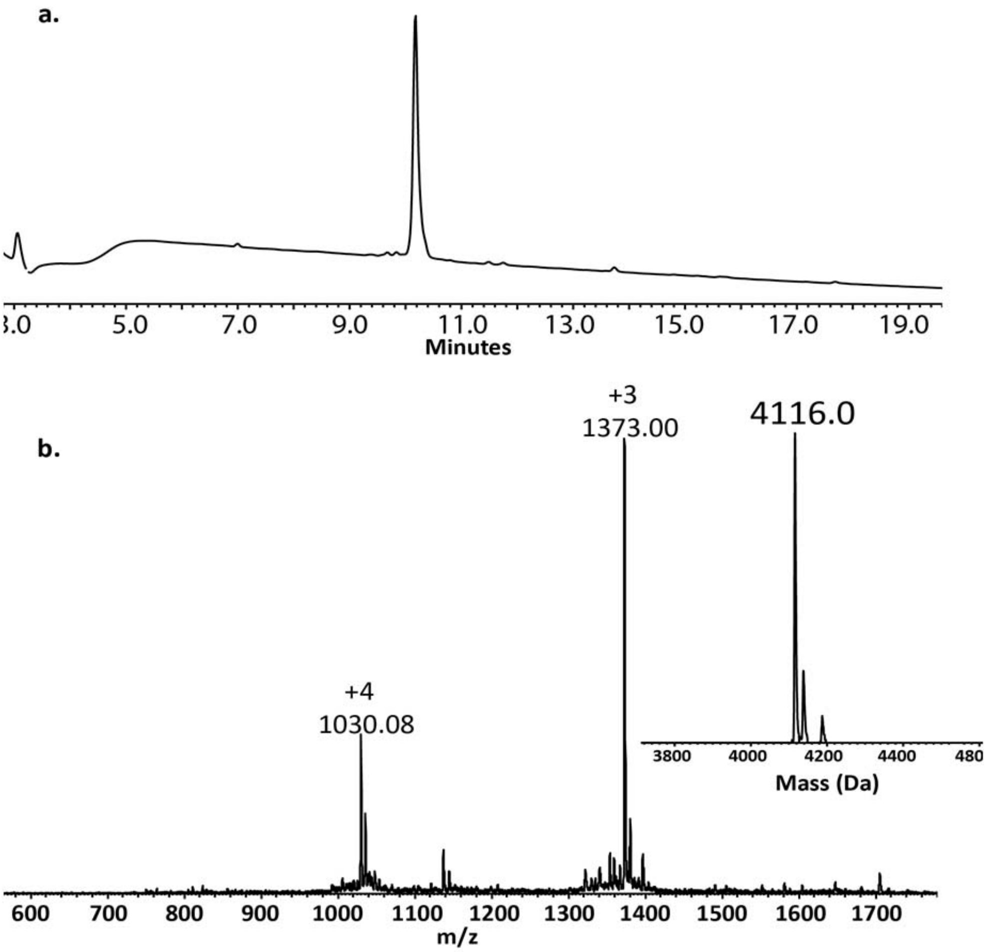

Figure S6. Characterization of Hir(1-38)-SR segment. **a**. Analytical HPLC analysis of purified Hir(1-38)-COSR (220 nm); **b**. The corresponding ESI-MS, with its deconvoluted mass (inset) (obs. average 4116.0 ± 0.2 Da, calc. Da 4115.5). The peptide was purified with five reduced thiols.

Non-native Hir(1-38)(C6U/C16U)-Nbz

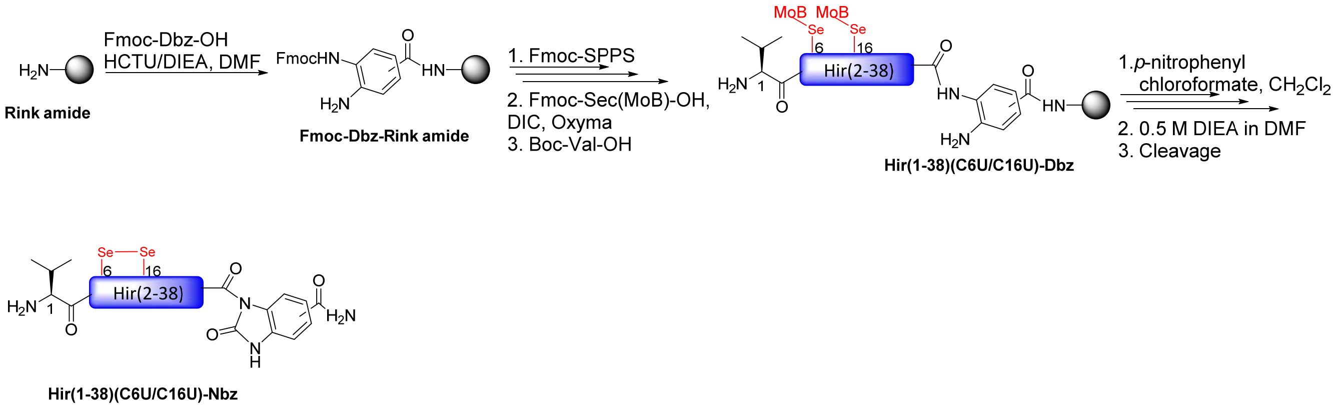

Scheme S4. Total chemical synthesis of Hir(1-38)(C6U/C16U)-Nbz by SPPS

The non-native diselenide-containing analog Hir(1-38)(C6U/C16U)-Nbz was synthesised on Fmoc-Dbz-resin.[7] 0.25 mmol scale of TentaGel Ram resin (0.19 g) was used for the synthesis. Mono-Fmoc-3,4-diaminobenzoic acid (Fmoc-Dbz-OH, 3 equiv) was activated with HCTU (3 equiv)/DIEA (6 equiv) in DMF and coupled manually to the free amine of the resin for 2 h. The synthesis was completed on an automated peptide synthesizer. The N-terminal amino acid was coupled manually as Boc-Val-OH (3 equiv) using HCTU (3 equiv)/DIEA (3 equiv) in DMF. Gly34 was manually coupled as (Dmb)Gly (2.5 equiv Fmoc-(Dmb)Gly-OH[2], 2.5 equiv HCTU and 5 equiv DIEA) and Asp33 was double coupled. Sec6 and Sec16 were manually coupled for 2 h each (3.0 equiv Fmoc-Sec(Mob)-OH activated on ice for 5 min using 3.0 equiv OxymaPure and 2.9 equiv DIC). The cleavage was done in the presence of 2 equiv of DTNP,[5] using TFA:water:thioanisole (95:2.5:2.5) cocktail for 3-4 h.

<u>On resin Nbz formation</u> was performed by treating the peptide-Dbz-resin with a solution of of *p*-nitrophenyl chloroformate (5 equiv) in DCM and shaken for 1 h at room temperature. Following this, the resin was washed and a solution of 0.5 M DIEA in DMF and shaken for additional 1 h to complete Nbz formation (repeated twice). Finally, the peptide-resin was washed using DCM and dried under vacuum.[7] The peptide was cleaved using TFA:trisopropylsilane(TIPS):water (95:2.5:2.5) for 3 h. Purification by RP-HPLC (C18 column) yielded ~3 % of pure peptide (Scheme S4, Figure S7).

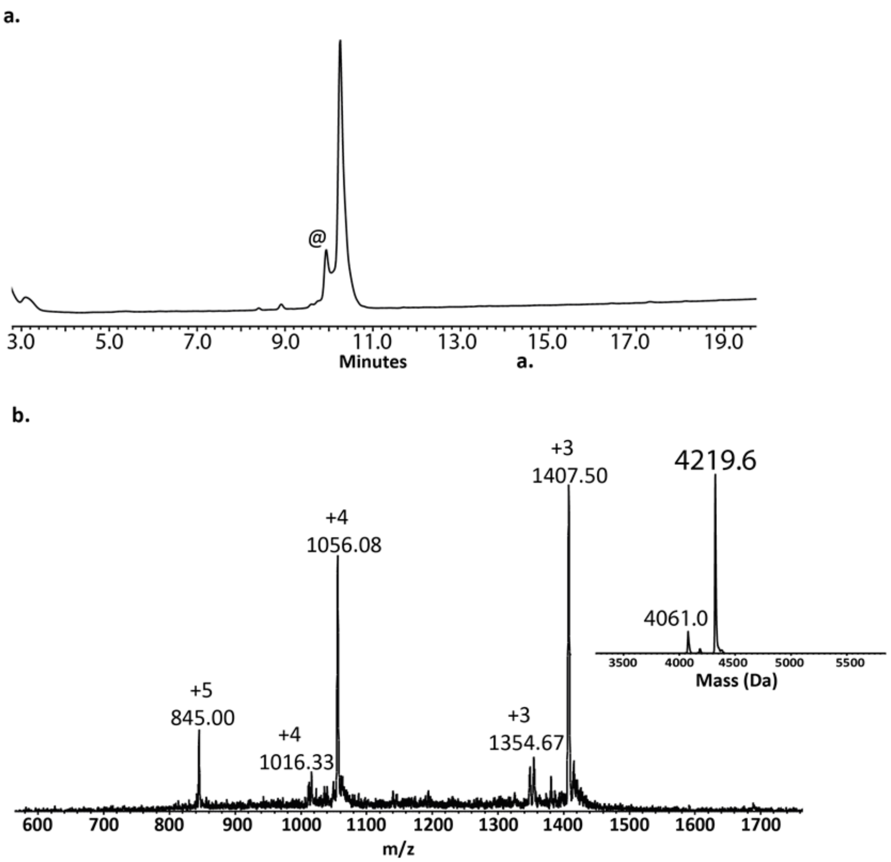

Figure S7. Characterization of the Hir(1-38)(C6U/C16U)-Nbz segment. **a**. Analytical HPLC analysis of purified Hir(1-38)(C6U/C16U)-Nbz peptide (220 nm); **b**. The corresponding ESI-MS with its deconvoluted mass (inset) (obs. average 4219.6 ± 0.2 Da, calc. Da 4219.3). The peptide was purified with single diselenide crosslink and three reduced thiols. @ hydrolysis side reaction of Hir(1-38)(C6U/C16U)-Nbz.

4.2. Native chemical ligations (NCL)

General Note: It is worth noting that Sec residues are prone to oxidation, forming either selenylsulfides or diselenide bonds. Mass analyses for all Se-Hir analogues indicated the formation of a single crosslink after NCL and HPLC purification, suggesting the formation of a single diselenide bond and four free thiols.

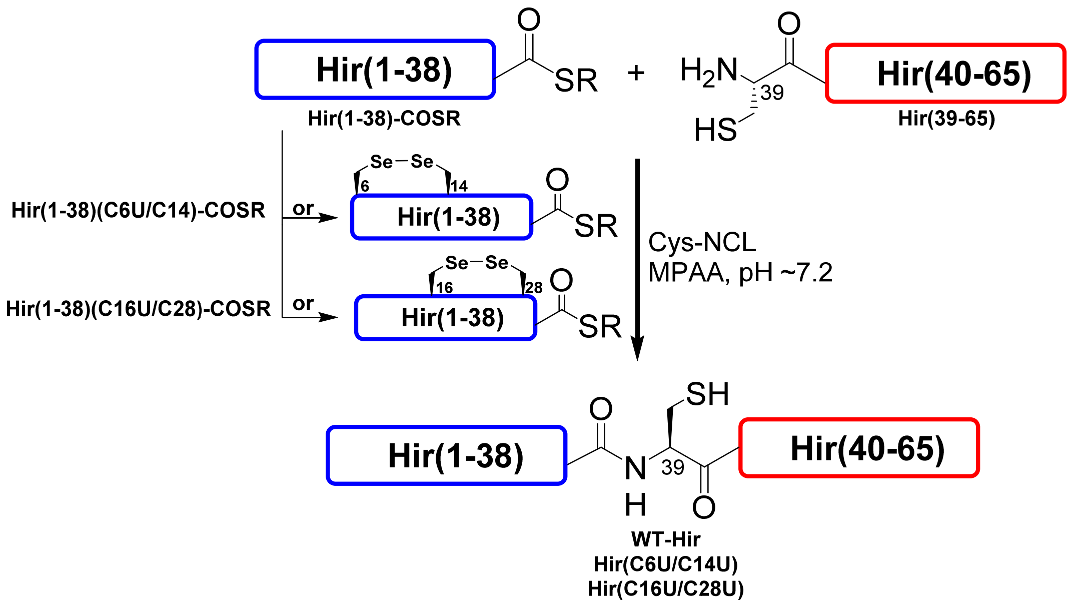

Scheme S5. Total chemical synthesis of WT-Hir, Hir(C6U/C14U) and Hir(C16U/C28U) by native chemical ligation (NCL)

WT-Hir. Hir(39-65) peptide (5.3 mg, 1.76 µmol, ~3 mM) was dissolved in 0.48 mL of argon degassed buffer (200 mM PB, 6 M Gn·HCl, 0.2 M MPAA, pH 7.2) and this mixture was added to Hir(1-38)-COSR peptide (~10 mg, 0.95 µmol, ~3 mM). The reaction was followed by analytical HPLC (C4 column), and completed in 18 h. The product was purified by semi-prep (C4 column) to afford ~52% (~8 mg) of pure protein (Scheme S5, Figure S8).

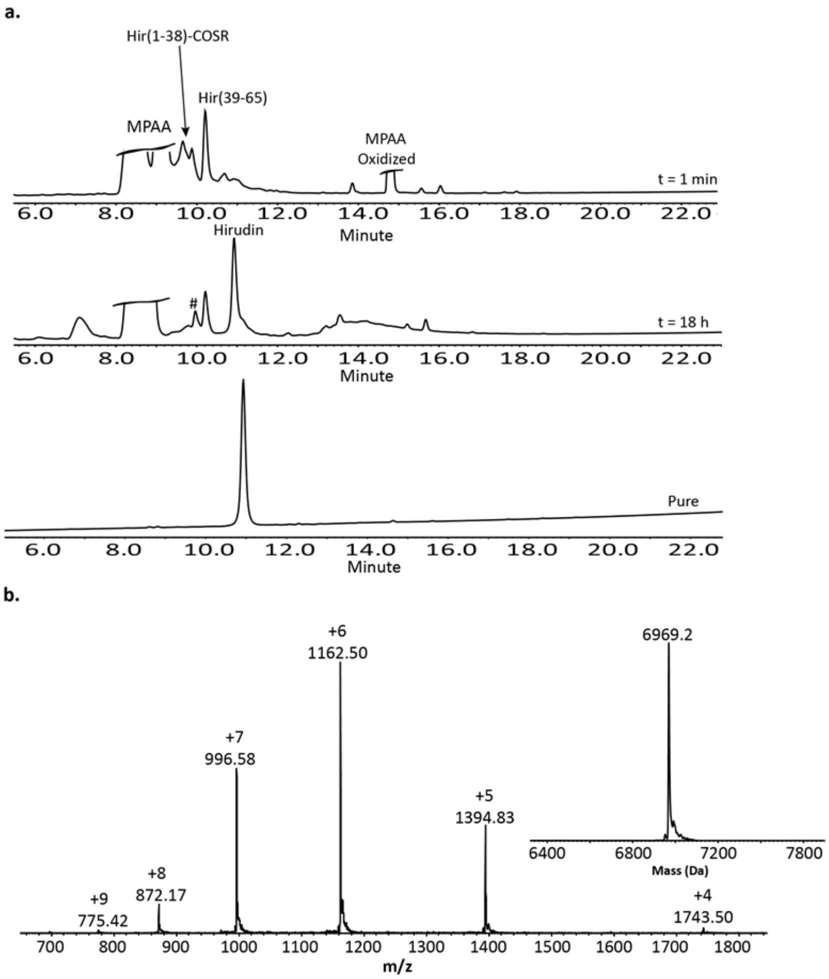

Figure S8. Preparation of WT-Hir. **a**. Analytical HPLC of Cys-NCL reaction (220 nm). The ligated product purified by semi-prep HPLC. **b**. The corresponding ESI-MS with four reduced thiols (obs. average 6969.2 ± 0.5 Da, calc. 6969.5 Da). # hydrolysis side reaction of Hir(1-38)-COSR.

Hir(C6U/C14U). Hir(39-65) peptide (6 mg, 1.99 µmol, ~3 mM) was dissolved in 0.66 mL of argon degassed buffer (200 mM PB, 6 M Gn·HCl, 0.2 M MPAA, pH 7.2) and this mixture was added to Hir(1-38)(C6U/C14U)-COSR peptide (~7 mg, 1.66 µmol, ~3 mM). The reaction was followed by analytical HPLC (C4 column), and completed in 18 h. The product was purified by semi-prep (C4 column) to afford ~45% (~5.8 mg) of pure protein (Scheme S5, Figure S9).

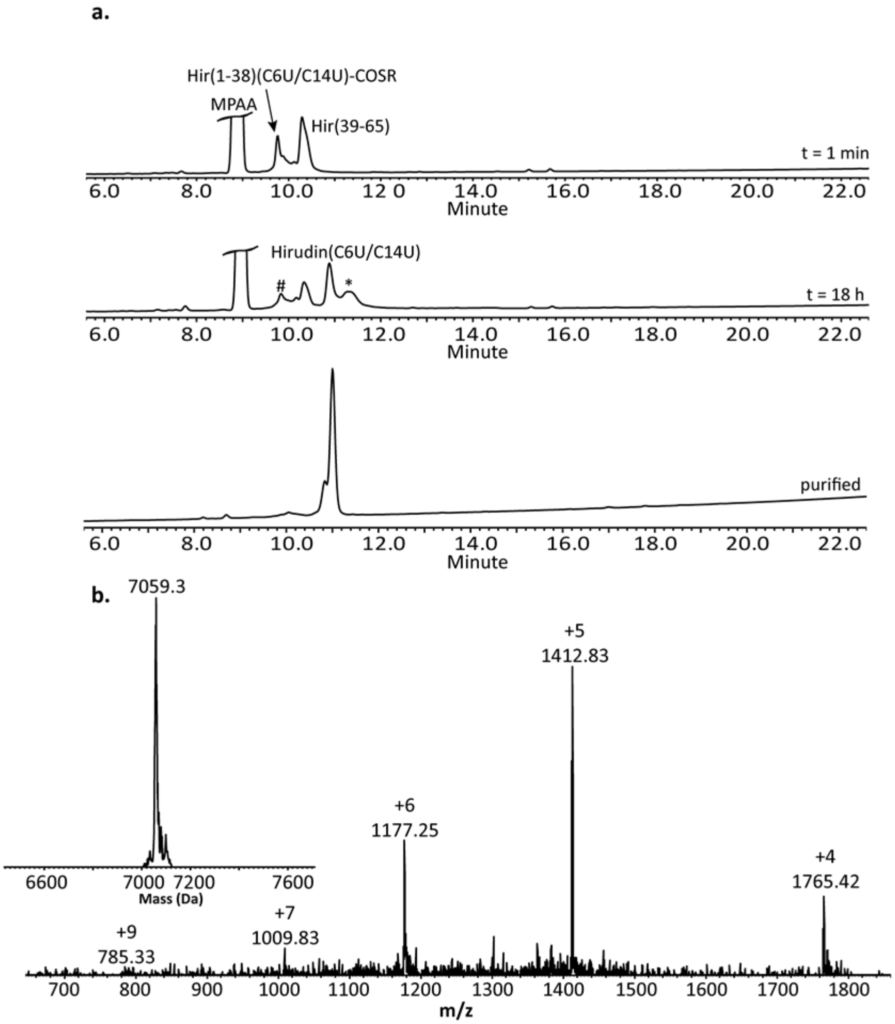

Figure S9. Preparation of Hir(C6U/C14U). **a**. Analytical HPLC of Cys-NCL reaction (220 nm). The ligated product purified by semi-prep HPLC. **b**. The corresponding ESI-MS of Hir(C6U/C14U) oxidized with a diselenide at position 6–14 and four reduced thiols. (obs. average 7061.0 ± 1.8 Da, calc. 7061.2 Da). # hydrolysis side reaction of Hir(1-38)(C6U/C14U)-COSR.

Hir(C16U/C28U). Hir(28-65) peptide (7 mg, 2.35 μmol, ~3 mM) was dissolved in 0.78 mL of argon degassed buffer (200 mM PB, 6 M Gn·HCl, 0.2 M MPAA, pH 7.2) and this mixture was added to Hir(1-38)(C16U/C28U)-COSR peptide (~9 mg, 2.14 μmol, ~3 mM). The reaction was followed by analytical HPLC (C4 column), and completed in 18 h. The product was purified by semi-prep (C4 column) to afford ~47% (~7.5 mg) of pure protein (Scheme S5, Figure S10).

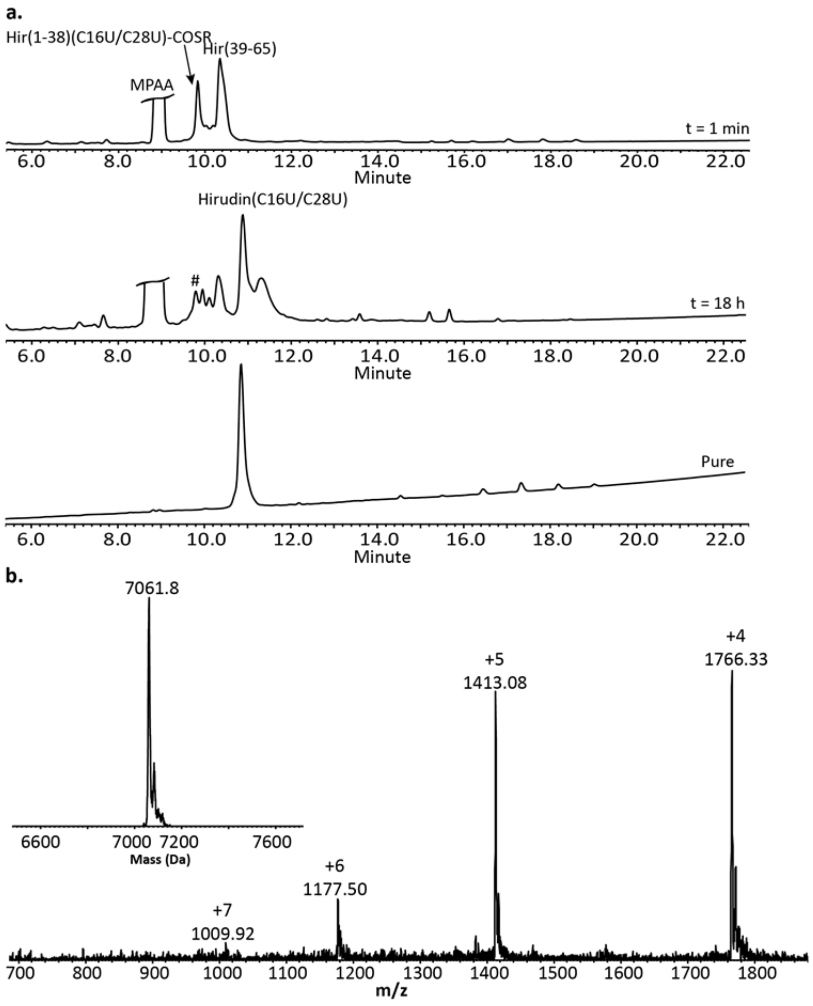

Figure S10. Preparation of Hirudin(C16U/C28U). **a**. Analytical HPLC of Cys-NCL reaction (220 nm). The ligated product purified by semi-prep HPLC; **b**. ESI-MS of Hirudin(C16U/C28U) oxidized with diselenide bond and four reduced thiols. (obs. average 7061.8 ± 1.4 Da, calc. 7061.2 Da). # hydrolysis side reaction of Hir(1-38)(C16U/C28U)-COSR.

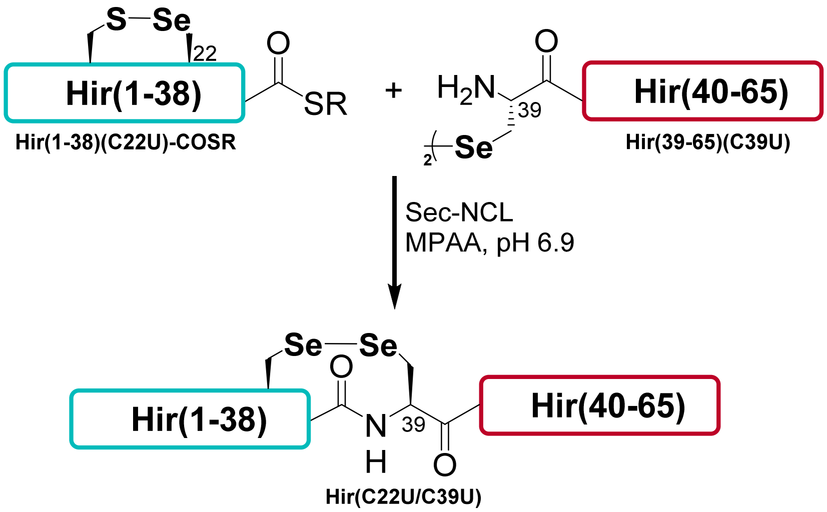

Scheme S6. Total chemical synthesis of Hir(C22U/C39U) by NCL

Hir(C22U/C39U). Hir(39-65)(C39U) peptide (3.7 mg, 1.2 µmol, ~3 mM) was dissolved in 0.4 mL of argon degassed buffer (200 mM PB, 6 M Gn·HCl, 0.2 M MPAA, pH 6.9) and this mixture was added to Hir(1-38)(C22U)-COSR peptide (~5 mg, 1.2 µmol, ~3 mM). The reaction was followed by analytical HPLC (C4 column), and completed in 18 h. The product was purified by semi-prep (C4 column) to afford ~17% (~1.5 mg) of pure protein (Scheme S6, Figure S11).

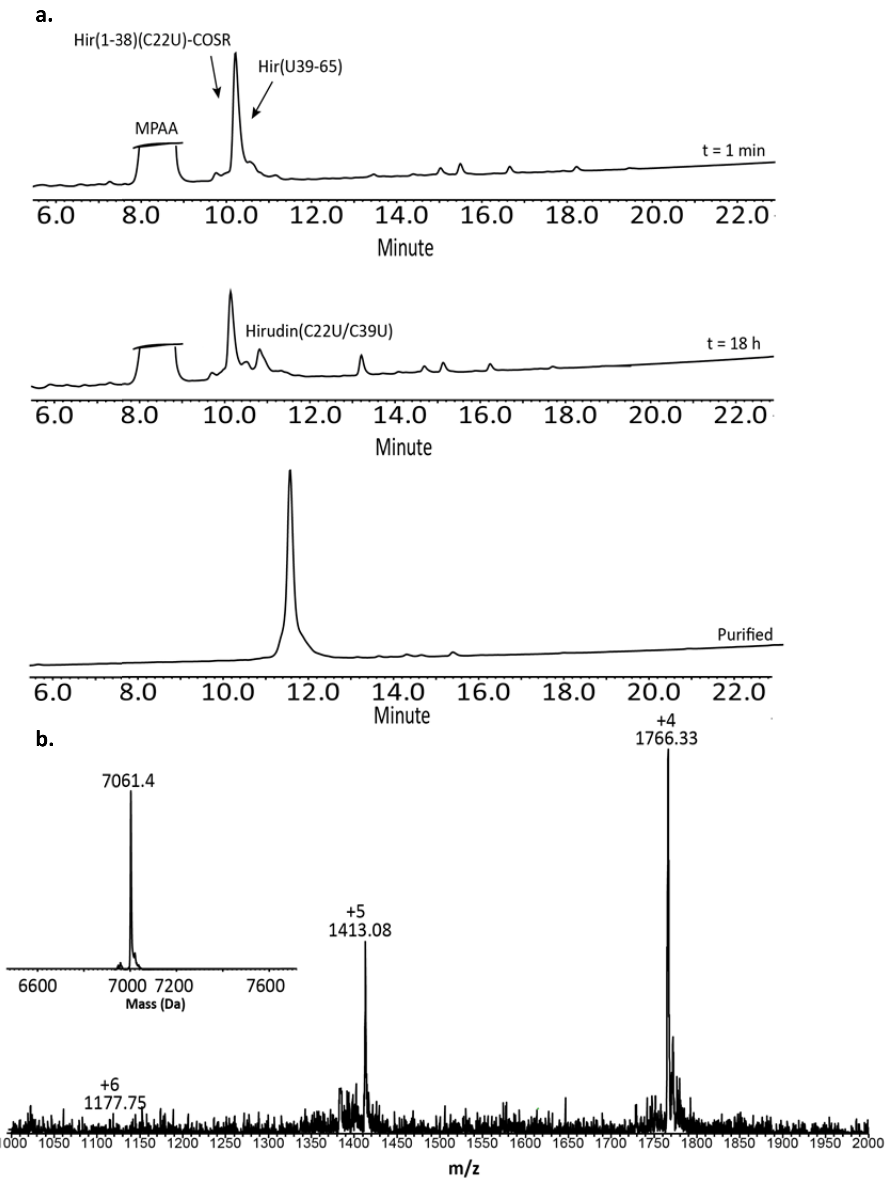

Figure S11. Preparation of Hir(C22U/C39U). **a**. Analytical HPLC of Sec-NCL reaction (220 nm). The ligated product purified by semi-prep HPLC. **b**. The corresponding ESI-MS of Hir(C22U/C39U) oxidized with diselenide bond and four reduced thiols. (obs. average 7061.4 ± 1.4 Da, calc. 7061.2 Da).

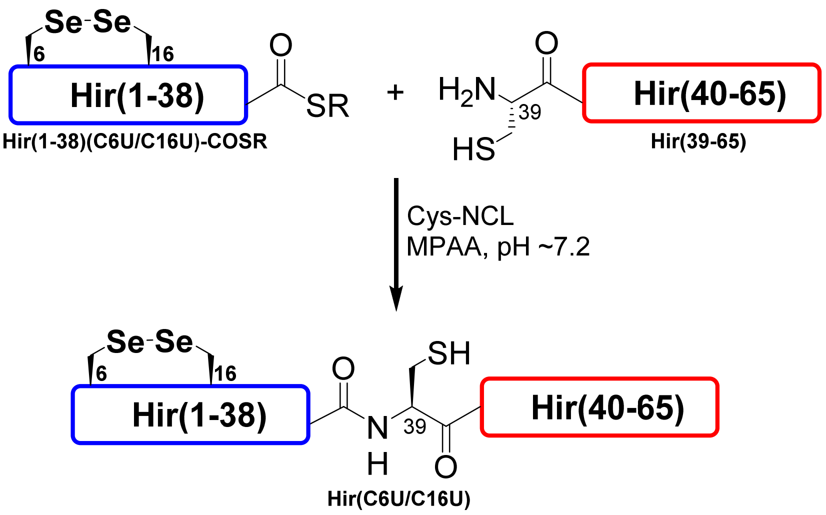

Scheme S7. Total chemical synthesis of, Hir(C6U/C16U) by NCL

Hir(C6U/C16U). The Hir(39-65) peptide (4.3 mg, 1.43 µmol, ~3 mM) was dissolved in 0.48 mL of argon degassed buffer (200 mM PB, 6 M Gn·HCl, 0.2 M MPAA, pH 7.2) and this mixture was added to Hir(1-38)(C6U/C16U)-Nbz peptide (~4 mg, 0.95 µmol, ~3 mM). The reaction was followed by analytical HPLC (C4 column), and completed in 10 h. The product was purified by semi-prep (C8 column) to afford ~37% (~3 mg) of pure peptide (Scheme S7, Figure S12).

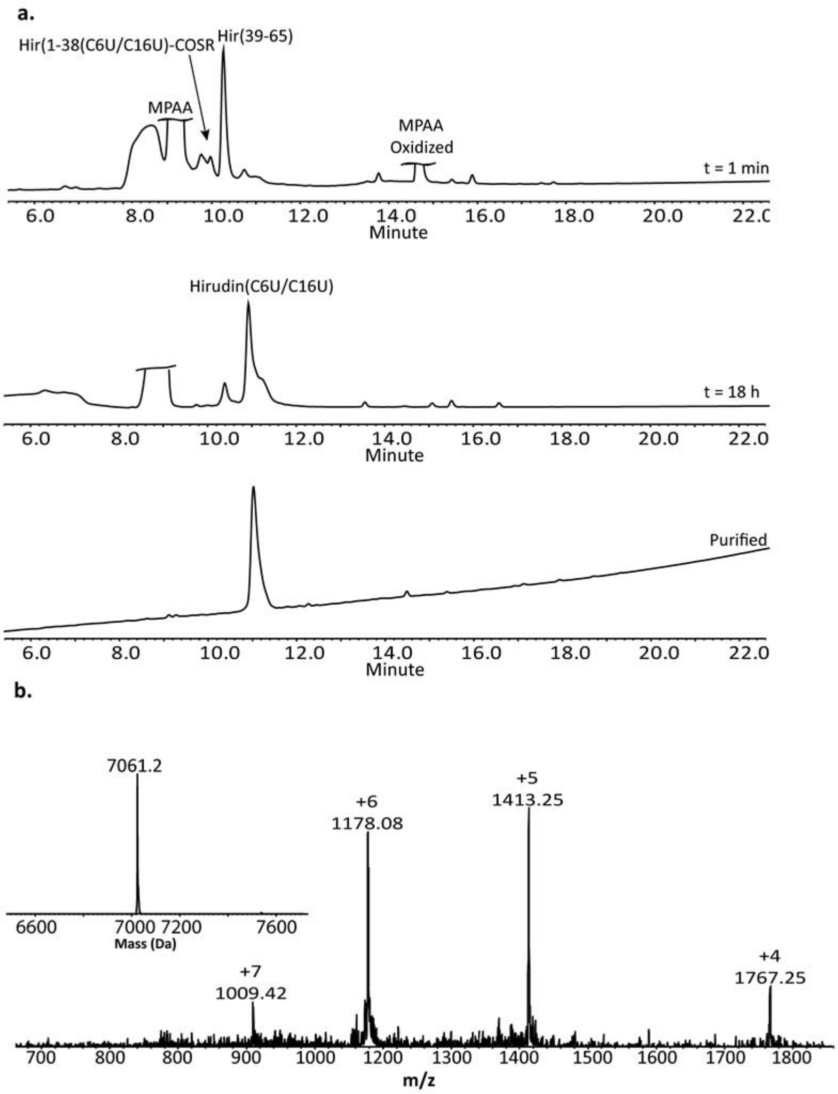

Figure S12. Preparation of Hir(C6U/C16U). a. Analytical HPLC of Cys-NCL reaction (220 nm). The ligated product purified by semi-prep HPLC. b. The corresponding ESI-MS of Hir(C6U/C16U) oxidized with diselenide bond and four reduced thiols. (obs. average 7061.2 ± 0.7 Da, calc. 7061.2 Da).

4.3. Oxidative folding

The oxidative folding experiments were performed according to the reported study by Chang *et al*, to allow for a direct comparison.[8] All folding reactions were performed under anaerobic conditions (except if stated otherwise) in an anaerobic chamber (Coy Laboratories Inc., O_2 sensor kept at <5 ppm) with nitrogen and hydrogen atmosphere (95%:5%) in degassed Tris·HCl buffer (100 mM Tris·HCl, 200 mM NaCl, 1mM EDTA, pH 8.7). Oxidized glutathione (GSSG, 5 equiv, final concentration 150 µM was added to 30 µM of reduced WT-Hir and its seleno-analogs. At various time intervals, 80 µL aliquots were removed and quenched with 30 µL of 2 M HCl, and stored at -20°C before analysis by analytical HPLC. The reaction mixture was injected into Atlantis T3 column (3 µm, 4.6 × 150 mm heated to 40 °C) and eluted from the column by 15:85 to 22:78 gradient over 15 min (B:A), and increasing to 28:72 over 32 min, and reaching the initial conditions over 36 min. All chromatograms were monitored at a wavelength of 220 nm (Fig. 2 in the main text).

The same experiment was repeated under aerobic conditions and in the absence of GSSG with WT-Hir and Hir(C16U/C28U) (Fig. S13 and Fig. 3a in the main text), in which oxygen is the only oxidant in solution. The folding was much slower compared to anaerobic conditions and in the presence of GSSG.

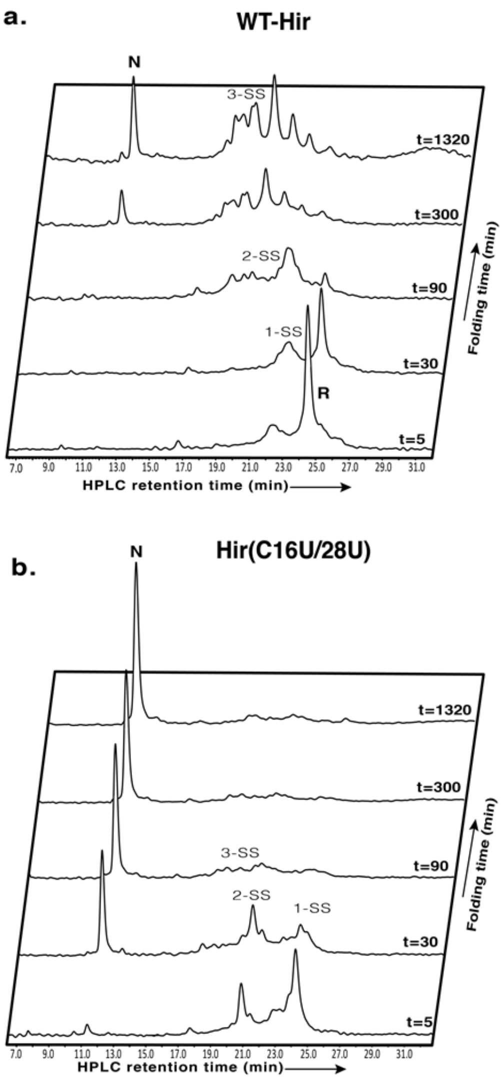

Figure S13. The aerobic oxidative folding at pH 8.7 and in the absence of GSSG for 30 µM of (**a**) WT-Hir and (**b**) Hir(C16U/C28U). 1-SS, 2-SS and 3-SS represents the number of disulfide (or diselenide) crosslinks in the intermediates.

4.4. X-Ray crystallography: Crystallization, data collection and structural analysis

The lyophilized Se-hirudin (Se-Hir) protein analogs were dissolved in water to a final concentration of 10 mg/mL. Thrombin from bovine plasma (Sigma-Aldrich) was dissolved in a solution containing 0.08 M sodium phosphate buffer (pH 7.5), 0.5 M NaCl and 0.1% PEG 6000 to prepare an 8 mg/mL protein solution. The three Se-Hir analogs, Hir(C16U/C28U); Hir(C6U/C14U); Hir(C22U/C39U), prepared in this way were then mixed with the thrombin solution (in molar ratio of 1:1.3 for Thrombin/Se-Hir analog) to obtain the three Se-Hir-Thrombin complexes submitted for crystallization.

4.4.1. The Hir(C16U/C28U)-Thrombin complex (Complex-1)

Crystals of the Hir(C16U/C28U)-Thrombin complex were obtained in about one day by the hanging-drop vapor diffusion method. Crystallization drops contained 1 μL complex and 1μL reservoir solution, equilibrated over 500 μL of reservoir solution, containing 38% PEG 4000, 0.1 M sodium phosphate (pH 4.7), and 0.2 M NaCl. Streak seeding was performed using 1/100 micro-seed stock solution. The crystals were prepared for data collection by flash freezing in liquid nitrogen directly, without any additional cryogenic solution. X-ray diffraction data were collected at the Se-peak wavelength (λ= 0.97864 Å) using the P13 beamline of the DESY synchrotron facility (Hamburg, Germany), indicating that these crystals belong to the $C222_1$ space group. The datasets were processed and integrated using the *DIALS* software package,[9] reduced (scaled and merged) by *Aimless*[10] (provided within the *CCP4i2*-graphical user interface of the *CCP4* software suit)[11] to a final resolution of 1.6Å. Molecular replacement structure determination was performed by *Phenix.phaser*[12], using the PDB-deposited structure of WT-Hir-thrombin (bovine) complex (PDB ID: 1HRT[13]) as a search model. The initial structure was adjusted and improved by manual building with *Coot*[14] and refined using *Refmac5*[15] (within the *CCP4i2*-graphical user interface)[11], resulting in a final R_{work} of 0.182 and R_{free} of 0.216 (Table S1).

4.4.2. The Hir(C6U/C14U)-Thrombin complex (Complex-2)

Crystals of the Hir(C6U/C14U)-Thrombin complex were obtained roughly by the same procedure described for Complex-1 above within 1-2 days of equilibration. X-ray diffraction data were collected remotely at the Se-peak wavelength (λ= 0.9763 Å) using the I04 beamline of the Diamond light source (London, UK), as a part of a CCP4/BGU workshop on "Advanced methods for macromolecular structure determination". The dataset, confirming that these crystals also belong to the $C222_1$ space group, were automatically processed using *Xia2-Dials* at the beamline working station, to a final resolution of 1.9 Å. Molecular replacement was performed by by *Phaser* (CCP4i2-graphical user interface of CCP4 program suit)[11] using the structure of Complex-1 as a search model. The initial structure was adjusted and improved by manual building with *Coot*[14] and refined by *Refmac5*[15], resulting in a final R_{work} of 0.214 and R_{free} of 0.263 (Table S1).

4.4.3. The Hir(C22U/C39U)-Thrombin complex (Complex-3)

Crystals of Hir(C22U/C39U)-Thrombin complex were obtained by a similar procedure to that described above for Complex-1 and Complex-2, except that the crystallization condition contained 0.3 M NaCl and the streaked seeding was performed with 1/10 micro-seeds solution. X-ray diffraction data were collected at the Se-peak wavelength (λ= 0.97864 Å) using the P11 beamline of the DESY synchrotron facility (Hamburg, Germany). The dataset, confirming again that these crystals belong to the $C222_1$ space group, were automatically processed using the *XDS* software package[16], reduced and merged by *Aimless*[10] to a final resolution of 2.7 Å. A molecular replacement was performed by *Phaser* (CCP4i2-graphical user interface of CCP4 program suit)[11] using the structure of Complex-1 as a search model. The initial structure obtained was further improved by manual building with *Coot*[14] and refined by *Refmac5* using restrained refinement[15], resulting in a final R_{work} of 0.194 and R_{free} of 0.253. Geometric validation for the three final structures of Complexes 1-3 were performed by *Molprobity*[17]and using the validation tools provided in *Coot*[5]. Data collection and refinement statistics for the three structures are summarized in **Table S1**.

Table S1. Crystallographic data collection and refinement parameters for the three Se-Hir-Thrombin complexes (Complex-1, Complex-2, Complex-3)

* data in parentheses refer to the last resolution shell

** No. of reflections used to calculate R_{free} (about 5% of the total)

	**Hir(C16U/C28U)-Thrombin**	**Hir(C6U/C14U)-Thrombin**	**Hir(C22U/C39U)-Thrombin**
Data Collection			
X-ray source	P13-DESY	I04-Diamond	P11-DESY
Wavelength (Å)	0.9786	0.9763	0.9778
Space group	$C222_1$	$C222_1$	$C222_1$
Cell dimensions			
a, b, c (Å)	58.67, 102.37, 142.74	58.10, 101.99, 144.04	58.07, 101.50, 142.07
α, β, γ (°)	90, 90, 90	90, 90, 90	90, 90, 90
Resolution (Å) *	48.18-1.60 (1.66-1.60)	72.02-1.90 (1.93-1.90)	47.79-2.70 (2.83-2.70)
R_{merge}	0.096 (2.37)	0.206 (1.848)	0.287 (1.80)
R_{pim}	0.028 (0.693)	0.062 (1.061)	0.082 (0.514)
R_{meas}	0.1 (2.470)	0.216 (2.148)	0.299 (1.873)
<I/σ(I)>	13.8 (1.1)	5.0 (0.2)	8.9 (1.6)
Completeness (%)	99.9 (98.3)	98.1 (78.8)	100 (99.9)
Redundancy	13.0 (12.5)	10.6 (4.09)	13.1 (13.1)
$CC_{1/2}$	0.999 (0.421)	0.992 (0.873)	0.992 (0.592)
Refinement			
Resolution (Å)	48.23-1.60	72.13-1.90	47.84-2.70
No. of reflections			
all	56953	33424	11902
for R_{free}**	2797	1572	586
R_{work} / R_{free}	0.182/ 0.216	0.214/0.263	0.194/0.253
No. of atoms			
Protein	2809	2850	2836

Ligand/ion	40/1	26/1	26/1
Water	357	215	73
B-factors <B_{fact}>			
Protein	31.61	40.92	45.45
Ligand/ion	77.01/42.8	55.93/29.67	61.99/28.2
Water	42.8	43.29	36.41
R.M.S.D.			
Bond length (Å)	0.0114	0.0061	0.0100
Bond angles (^{o})	1.875	1.453	1.744
PDB code	7A0D	7A0E	7A0F

4.4.4. Supplementary discussion- Further structural analysis

4.4.4.1. The overall structures

As described in the main text, three of the disulfide bonds of WT-Hir have been replaced with diselenide bonds (at crosslinks 16–28, 6–14, and 22–39), one in each of the three Se-Hir analogs discussed here. The three Se-Hir derivatives were then mixed with their target thrombin protease (from a bovine source) to form tight and stable Se-Hir-Thrombin complexes; the Hir(C16U/C28U)-Thrombin complex (Complex-1), the Hir(C6U/C14U)-Thrombin complex (Complex-2), and the Hir(C22U/C39U)-Thrombin complex (Complex 3). These complexes were subjected to comprehensive structural analysis by X-ray crystallography, to dissect any differences observed between the three Se-Hir analogs. These final 3D structures are presented in the main manuscript (**Figure 4**), and the corresponding structural determination parameters are listed in **Table S1**. As demonstrated in this table, although the three complexes were determined at different resolutions, their final crystallographic structural parameters are quite good, confirming that these structures are sufficiently reliable for detailed analyses and comparisons, as further discussed below. In this respect, it is noted that several side chains have not been modeled in the final structures of the three Se-Hir-Thrombin complexes presented here, mainly due to ambiguous electron density, perhaps originating from the local flexibility of these side chains. This is specifically the case with the thrombin side chains of tLeu64 (chain I), tTyr71 (chain H) and tGlu72 (chain H) in Complex-1, and the side chain of hGln65 (chain I) in both Complex-1 and Complex-2. These side chains are therefore not included in the structural analysis and comparisons discussed below. In order to keep the structural analysis clear and simple, we used a prefix of "t" and "h" to refer to residues of the thrombin and hirudin, respectively. Yet, to remain consistent with previous reports related to thrombin/hirudin structures, we also kept in some places in the text and the tables the postfix labeling of the commonly used PDB chains, using "L" for the light chain of thrombin, "H" for the heavy chain of thrombin, and "I" for the entire chain of the hirudin inhibitor. These postfix labels of L, H, and I were also used in the deposited PDB coordinates of the current structures.

In general, all three structures are quite similar to each other, and to the reported 3D structure of WT-Hir in complex with bovine thrombin (PDB ID: 1HRT).[13] A global structural comparison of the bound Se-Hir in Complex-1-3, to the bound WT-Hir in this reference structure (PDB ID: 1HRT) gives relatively small (overall) RMSD values of 1.620, 1.476, 1.472 Å, respectively. These values, as well as a visual superposition of each of these pairs **(Figure S14)** indeed confirm that no major global

structural differences could be observed between the four structures compared, confirming that native folded states have been reached with all the protein analogs studied here. This led us to examine more carefully the local conformations at specific segments of the bound Se-Hir analog in these structures, in an attempt to identify some local structural changes, especially around the modified disulfide/diselenide bonds. These detailed local comparisons are further described below.

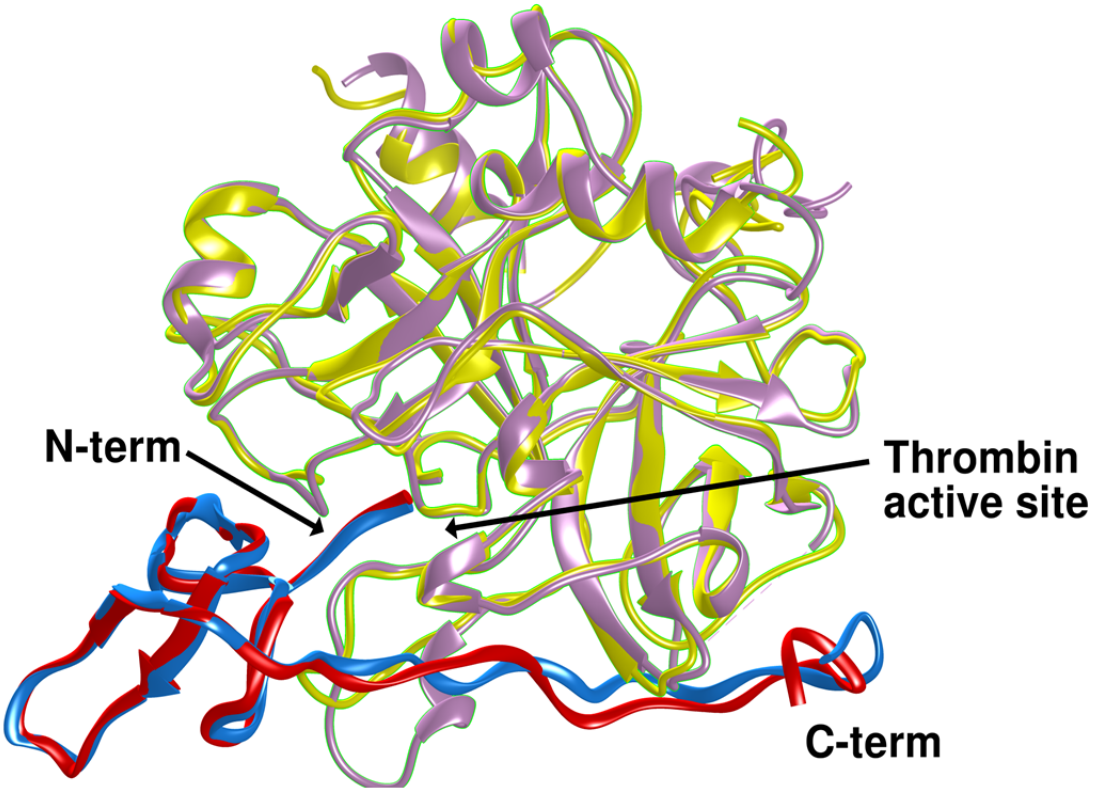

Figure S14. A superposition of the structure of Complex-1 with the corresponding reported structure of WT-Hir-Thrombin (PDB ID: 1HRT). Color codes: **Complex-1** - Thrombin heavy and light chains (*plum*); Hir(C16U/C28U) (*blue*, PDB ID: 7A0D). **PDB ID:1HRT** - Thrombin heavy and light chains (*yellow*); WT-Hir (*red*). The hirudin N-terminus and C-terminus are marked for both complexes.

4.4.4.2. The diselenide bonds and their environments

The Se-Se bond length in the three derivatives and the dihedral angles around them are listed (**Table S2**) and graphically presented (**Figure S15)**, compared to the corresponding disulfide bonds in WT-Hir in the published WT-Hir-Thrombin structure (PDB ID: 1HRT).[13]

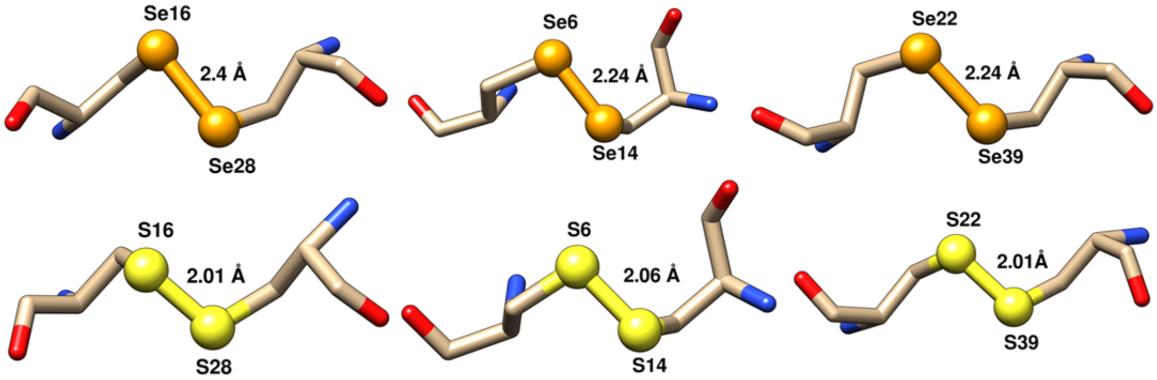

Figure S15. Comparison of the Se-Se bonds in the Se-Hir analogs analyzed here and the corresponding S-S bonds in the published structure of WT-Hir.

As expected, the diselenide bonds are longer than the corresponding disulfide bonds, in correlation to the larger atomic VDW cross-section of the interacting atoms. Interestingly, the diselenide bond in Hir(C16U/C28U) was found to be significantly longer compared to the other two diselenide bonds, yet still within the observed range of diselenide bond lengths. Surprisingly, however, the local environments of the three diselenide bonds are not considerably different from those of the corresponding disulfide bonds in the reported WT-Hir-thrombin complex. This is also the case with the unchanged disulfide bonds in the three current Se-Hir analogs.

Table S2. Dihedral angles around the three replaced Se-Se bonds in the current Se-Hir-Thrombin complexes, compared to the corresponding angles in the WT-Hir-Thrombin complex (PDB ID:1HRT).

	Hir(C16U/C28U)-Thrombin / PDB ID:1HRT		***Hir(C6U/C14U)-Thrombin / 1HRT***		***Hir(C22U/C39U)-Thrombin / 1HRT***	
Dihedral angels (°)	***Se16-Se28 / S16-S28***		***Se6-Se14 / S6-S14***		***Se22-Se39 / S22-S39***	
	Se16/S16	***Se28/S28***	***Se6/S6***	***Se14/S14***	***Se22/S22***	***Se39/S39***
Phi (φ)	-117/-91	-95 /-82	-55/-72	-157/-132	-79/-106	-79/-80
Psi (ψ)	-72 /-68	126/115	137/123	165/172	122/109	118/99
Omega (ω)	178. /-179	169/177.53	-175/178	-178/178	-172/-178	179/179
Chi (χ)	179/-170	-173/-167	-52/-53	48/59	-153/-179	-160/164

4.4.4.3. Intramolecular hydrogen bonds within the Se-Hir structures

We next examined the *intramolecular* hydrogen bonds within the Se-Hir structures, focusing mainly on identifying differences that would explain the disparate characteristics observed for the Hir(C6U/C14U) analog (in Complex-2), compared with WT-Hir, as well as to the other two Se-Hir derivatives. As noted in the past,[13] the hirudin protein contains a very stable core domain, built of residues 5-48, and contain three conserved disulfide bonds, from which two peptides are emanating away, a short peptide at the N-terminus (residues 1-4) and a longer peptide at the C-terminus (residues 49-65). The current structures of the three Se-Hir analogs demonstrate that the core domain of the protein remains practically unchanged, and is tightly held in its original conformation by a conserved network of intramolecular hydrogen bonds (**Figure S16**). These interactions are summarized in Table S3, together with a comparison to the corresponding interactions in the WT-Hir complex (PDB ID: 1HRT). In these analyses we use a relatively generous cutoff criterion for the distance (3.6Å) between the potential donors and acceptors of the hydrogen bonding interactions, in order to include as many interactions as possible. The presence of a potential hydrogen bond, and the relevant donor-acceptor distance, were determined using the commonly used routines in the *Chimera* software.[9] Using such analyses, we observed only relatively small differences in the hydrogen bond interactions amongst the four compared structures, and generally these differences do not seem to identify significant changes in molecular characteristics.

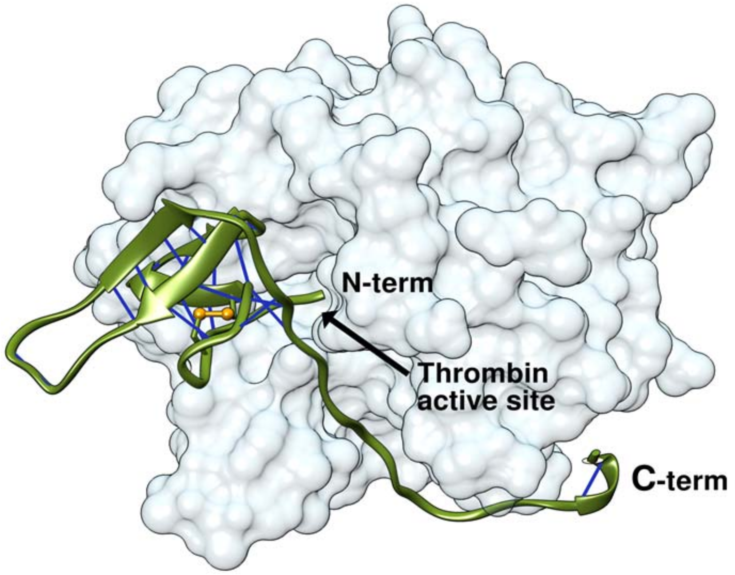

Figure S16. Hir(C6U/C14U) (*green*) as it appears in its tight complex with thrombin (Complex-2, PDB ID: 7A0E), demonstrating the tight binding of hirudin onto the specific grooves of thrombin. The Se-

Se bond is shown in *gold*, and the comprehensive network of intramolecular H-bonds (within hirudin) is shown in *blue*, accounting for the relatively rigid conformation of the core domain of this protein.

Table S3. Intramolecular hydrogen-bonding interactions within the current structures of the Se-Hir analogues as compared to the published structure of WT-Hir (PDB ID: 1HRT).

Hydrogen bonds/ amino acids		***Hir(C16U/C28U)***	***Hir(C6U/C14U)***	***Hir(C22U/C39U***	***WT-Hir***
Donor	*Acceptor*	*Distance (Å)* *D…A*	*Distance (Å)* *D…A*	*Distance (Å)* *D…A*	*Distance (Å)* *D…A*
Cys6.I N	Leu15.I O	2.966	------	------	------
Sec6.I N		------	3.011	------	------
Cys6.I N		------	------	3.002	------
Thr7.I N	Gln11.I OE1	2.767	2.685	2.706	3.077
Thr7.I OG1	Gln11.I OE1	3.454	------	------	------
Glu8.I N	Gln11.I OE1	3.406	------	------	------
Gly10.I N	Sec28.I O	2.738	------	------	------
	Cys28.I O	------	2.930	3.116	2.937
Gln11.I NE2	Thr7.I OG1	3.317	3.042	------	3.171
Gln11.I N	Glu8.I O	3.287	------	------	------
Asn12.I N	Thr45.I O	2.881	2.802	2.711	2.759
Asn12.I ND2	Gly23.I O	------	3.468	3.043	------
Asn12.I ND2	Asn26.I O	2.977	2.929	2.813	2.880
Asn12.I ND2	Gly44.I O	------	------	3.424	------
Leu13.I N	Cys22.I O	2.806	2.666	------	------
	Sec22.I O	------	------	2.697	------
Leu15.I N	Thr4.I O	2.741	2.689	2.908	2.814
Sec16.I N	Asn20.I O	3.090	------	------	------
Cys16.I N					

		------	3.206	3.099	3.197
Asn20.I N	Glu17.I O	3.203	3.085	2.958	------
Asn20.I ND2	Glu17.I O	2.894	2.806	2.711	------
Cys22.I N	Cys14.I O	2.862	------	------	------
Cys22.I N	Sec14.I O	------	2.820	------	------
Sec22.I N	Cys14.I O	------	------	2.675	------
Cys22.I N	Cys14.I O	------	------	------	2.467
Asn26.I N	Gly23.I O	3.098	3.173	------	------
Asn26.I ND2	Gly23.I O	3.307	3.439	------	------
Asn26.I ND2	Gln24.I O	3.332	3.066	------	------
Lys27.I N	Val40.I O	2.799	2.734	2.676	2.713
Lys27.I NZ	Thr41.I O	------	------	------	2.984
Sec28.I N	Gln11.I O	2.932	------	------	------
Cys28.I N		------	2.955	2.925	2.937
Ile29.I N	Gln38.I O	2.743	2.671	2.721	2.947
Leu30.I N	Ser9.I OG	2.858	2.859	3.052	------
Gly31.I N	Asn37.I OD1	2.791	2.776	2.854	3.401
Ser32.I N	Glu35.I OE2	2.589	2.907	3.264	------
Ser32.I OG	Glu35.I OE2	3.188	2.634	------	------
Glu35.I N	Ser32.I O	------	------	------	3.139
Glu35.I N	Ser32.I O	2.908	3.025	------	------
Asn37.I ND2	Sec16.I O	2.980	------	------	------
	Cys16.I O	------	3.157	2.971	3.486
Gln38.I N	Ile29.I O	3.027	2.960	3.017	2.942
Cys39.I N	Glu17.I OE2	2.814	2.767	------	------

Sec39.I N		------	------	2.767	------
Val40.I N	Lys27.I O	2.932	2.735	2.758	2.764
Gly42.I N	Gly25.I O	2.944	2.988	2.920	2.998
Thr45.I N	Gly10.I O	2.930	2.905	2.710	------
Lys47.I N	Asn12.I O	2.942	2.998	3.025	------
Lys47.I NZ	Thr4.I OG1	2.764	2.499	2.626	3.090
Lys47.I NZ	Asp5.I O	2.726	2.999	2.769	2.588
Ser50.I N	Gln49.I OE1	------	------	------	3.042
Ser50.I OG	Trp45D.H O	------	------	------	3.485
Ser50.I OG	Gln49.I O	------	------	------	2.783
Asp53.I N	His51.I O	------	------	2.699	
Asp55.I N	Asp53.I OD1	2.980	------	------	------
Glu62.I N	Glu62.I OE1	2.811	------	------	------
Tyr63.I N	Pro60.I O	3.376	3.146	3.293	------
Leu64.I N	Glu61.I O	------	------	3.077	------
Gln65.I NE2	Gln65.I OXT	------	------	------	3.187
Gln65.I N	Glu61.I O	------	------	3.118	------

A closer look at this H-bonding analysis indicated that within the network that holds together the core of the hirudin molecule there is a specific "cluster" of several tight intramolecular hydrogen bonds, which seem to contribute the most to the robust conformation of this core (**Figure S17**). Interestingly, this H-bonding cluster is located all around the disulfide (or diselenide) bond between residues 6 and 14. This cluster includes mainly the hydrogen bonds between Thr4-Lys47, Asp5-Lys47, Thr7-Gln11, Asn12-Gly23, Asn12-Asn26, Asn12-Lys47 and Leu15-Thr4, which seem to be the most significant intramolecular interactions of the hirudin core. A focus on this central H-bonding cluster (in Complex-2) is shown in **Figure S17**, and a complementary comparison between these clusters in the Se-Hir structures of Complex-1 and Complex-2 is shown in Table S4, where the specific H-bonds and their

corresponding differences are listed. Both Figure S17 and Table S4 emphasize the central role of Lys47 in the stabilization of the core structure of the hirudin protein, as well as in holding its N-terminal and C-terminal emanating peptides in their specific directions. These results are reinforced by previous studies which showed that the hydrogen bonds formed by the side-chain amino group of Lys47 with the backbone carbonyl oxygen of Asp5 and the side-chain O atom of Thr4 help to position the N-terminal of hirudin in the active-site cleft.[18] Nevertheless, site directed mutagenesis experiments failed to demonstrate a crucial role for Lys47, or any of the other basic residues in the C-terminal peptide of hirudin on its inhibitory potency, since such inhibition was only slightly decreased when Lys47 was replaced with other amino acids.[18]

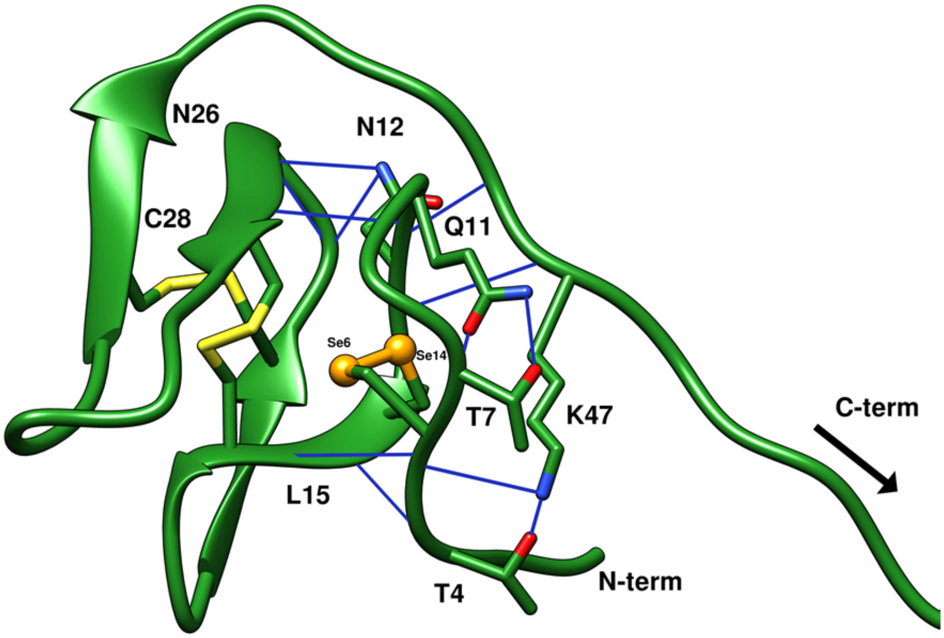

Figure S17. A close-up on the core of Hir(C6U/C14U), in its bound form with thrombin. The 6–14 diselenide bond is marked in *gold* and the cluster of H-bonds around it is shown in *blue*. Most of the Se-Hir residues participating in these H-bonds are labeled and their interacting side-chains are shown.

Table S4. Distances within the cluster of conserved hydrogen bonds in the cores of the Hir(C16U/C28U)-thrombin complex and Hir(C6U/C14U)-thrombin complex

Donor	Acceptor	Hir(C16U/C28U)	Hir(C6U/C14U)	Δ D1-D2 (Å)
		D--A (Å)	D--A (Å)	
Cys6.I N/Sec6	Leu15.I O	2.966	3.011	-0.045
Thr7.I N	Gln11.I OE1	2.767	2.685	0.082
Thr7.I OG1	Gln11.I OE1	3.454	3.415	0.039
Asn12.I N	Thr45.I O	2.881	2.802	0.079
Asn12.I ND2	Gly23.I O	3.783	3.468	0.315
Asn12.I ND2	Asn26.I O	2.977	2.929	0.048
Leu13.I N	Cys22.I O	2.806	2.666	0.140
Leu15.I N	Thr4.I O	2.741	2.689	0.052
Sec16.I N/ Cys16.I N	Asn20.I O	3.090	3.206	-0.116
Cys22.I N	Cys14.I O/Sec14.I O	2.862	2.820	0.042
Sec28.I N/ Cys28.I N	Gln11.I O	2.932	2.955	-0.023
Lys47.I N	Asn12.I O	2.942	2.998	-0.056
Lys47.I NZ	Thr4.I OG1	2.764	2.499	0.265
Lys47.I NZ	Asp5.I O	2.726	2.999	-0.273

4.4.4.4. Intermolecular hydrogen bonds between the Se-Hir analogs and Thrombin

We next analyzed the intermolecular hydrogen bonds that are involved in forming the current Se-Hir-Thrombin complexes, in an attempt to locate potential differences in the observed hirudin-thrombin interactions. As for the intramolecular H-bonding interactions within the Se-Hir analogs, the presence of a potential intermolecular hydrogen bond was determined with the *Chimera* software[19], using a relatively generous cutoff criterion for the distance (3.6 Å) between the potential donor and acceptor (**Table S5**).

Table S5. Intermolecular hydrogen-bonding interactions between thrombin and the bound Se-Hir analogs, compared to the corresponding interactions in the reported WT-Hir-thrombin complex (PDB ID: 1HRT)

Hydrogen bonds (amino acids)		***Hir(C16U/C28U) -Thrombin***	***Hir(C6U/C14U) -Thrombin***	***Hir(C22U/C39U) -Thrombin***	***WT-Hir-Thrombin***
Donor/Acceptor	*Acceptor/Acceptor*	*Distance (Å) D…A*	*Distance (Å) D…A*	*Distance (Å) D…A*	*Distance (Å) D…A*
Ser205.H OG (acceptor)	Val1.I N (donor)	3.122	-----	------	------
His43.H NE2 (acceptor)		-----	3.160	------	-----
His43.H NE2 (acceptor)		-----	-----	3.081	-----
Ser226.H O (acceptor)	Val1.I N (donor)	2.908	2.765	2.746	2.795
Gly228.H N	Val1.I O	3.159	3.260	3.401	2.735
Gly228.H O (acceptor)	Tyr3.I N (donor)	2.934	2.946	3.018	2.702
Gly230.H N	Tyr3.I O	2.844	2.773	2.814	------
Asn95.H OD1 (acceptor)	Tyr3.I OH (donor)	2.657	2.948	2.526	------
Arg233.H NH2	Asp5.I OD1	3.013	3.003	3.082	2.785
Arg178.H NH2	Glu17.I OE1	------	------	------	3.537
Arg233.H NH1	Ser19.I OG	2.885	3.059	3.232	------
	Asp5.I OD1	------	------	------	3.100
Lys236.H NZ	Ser19.I O	3.132	3.001	3.092	3.138
Lys236.H NZ	Asn20.I OD1	-----	------	-----	3.379
Glu229.H OE1 (acceptor)	Val21.I N (donor)	------	------	------	3.407
Glu229.H OE2 (acceptor)	Val21.I N (donor)	2.819	2.652	2.775	3.108
Trp50.H NE1	Lys47.I O	2.993	3.059	3.244	-----
Lys52.H NZ	Gln49.I OE1	3.196	------	------	------
Arg20.H NH2	Ser50.I O	------	------	------	3.520
Trp50.H O (acceptor)	Ser50.I OG (donor)	------	------	------	3.485
Glu25.H OE2 (acceptor)	Asn52.I ND2 (donor)	2.687	------	------	------

Asn143.H ND2	Asn52.I OD1	------	------	------	3.435
Leu26.H N	Asp53.I O	2.732	2.718	------	------
Arg68.H NH1	Asp53.I OD2	2.509	------	------	------
	Asp53.I OD1	------	------	------	2.524
Arg68.H NH2	Asp53.I OD1	2.949	------	------	------
Arg68.H NH2	Asp 53.I OD2	3.089	------	------	------
Arg68.H NH2	Asp55.I O	3.069	------	------	------
Gln24.H OE1 (acceptor)	Phe56.I N (donor)	-----	-----	-----	3.187
Tyr71.H N	Glu57.I OE2	-----	3.224	------	------
Gln24.H NE2	Ile59.I O	3.615	-----	------	------
Gln24.H OE1 (acceptor)	Ile59.I N (donor)	2.967	------	------	------

In general, the analysis showed that all three complexes (Complex-1-3) contained similar intermolecular hydrogen bonding patterns between the thrombin protein and the bound Se-Hir analogs, confirming the generally similar biological activities observed. Interestingly, however, the analysis indicated that the Hir(C16U/C28U) in Complex-1 forms slightly more hydrogen bonds compared to the other two derivatives, and especially those formed between Gln24.H and Ile59.I, Lys52.H and Gln49.I, Arg68.H and Asp53.I, Arg68.H and Asp55.I, and between Asn52.I and Glu25.H (where H refers to the heavy chain of thrombin, and I refers to the Se-Hir analog (Inhibitor) of complex-1). Obviously, it is possible that these additional H-bonds could be identified due to the higher resolution of Complex-1 (1.6 Å) compared to Complex-3 (2.7 Å), yet their systematic absence in Complex-2 (of the comparable resolution of 1.9 Å) may account, at least in part, for the slightly higher affinity to thrombin of Hir(C16U/C28U) as compared to Hir(C6U/C14U). This statement is supported by the fact that one of these additional H-bonds (Arg68.H to Asp53.I) is also identified in the thrombin complex of WT-Hir, which shows inhibition parameters similar to Hir(C16U/C28U).

Another intermolecular interaction between hirudin and thrombin that could be significant for both affinity and inhibition is that of the N-terminal residue, Val1, in hirudin. The positively charged

(terminal) amino group of this residue forms an important H-bond with specific residues in the active site of thrombin, thereby blocking the active site and contributing a critical factor to its inhibition. In all of the current complexes Val1 forms hydrogen bonds with Ser226 and Gly228 of thrombin. However, in Complex-1 it also forms a key H-bond with Ser205 of thrombin, the catalytic nucleophile of the hydrolytic reaction (related to the classical Ser195 in serine proteases). In Complex-2 this H-bond is not observed and is replaced with an alternative H-bond with His43, which is likely to be less important for inhibition. This finding is in correlation with previous observations in regards to WT-Hir, pointing out that the amino-terminus group of a bound hirudin forms H-bonds with the thrombin residues Ser205 and Ser226 (the equivalents for the classical residues Ser195 and Ser214), and as such presents the only positively-charged group of hirudin that interacts directly with the target thrombin. This role of Val1 was further reinforced by a removal of the positive charge of the amino-terminus moiety by its chemical acetylation, which reduced significantly the binding energy of the modified hirudin to thrombin (by ~23 kJ•mol^{-1}).[18]

4.4.4.5. RMS deviation by residue

Another form of comparison between the three current structures is to superimpose each pair of them and then analyze the RMS deviation (RMSD) per residue. Such analysis was done for the Se-Hir analogs in Complex-1 and Complex-2, being the most reliable structures at the highest resolution among the four related structures discussed here. This analysis is presented in Figure S18, where the plots of the RMSD-per-residue are based only on the Cα atoms of the residues, on the backbone atoms, or on all atoms of the residue (including the side chains). As expected, very small RMSDs are observed for the Cα's and the backbone atoms, confirming again that the two compared Se-Hir analogs are nearly identical in terms of the overall conformation and the secondary-structure elements, except for the obvious deviation observed for the flexible peptide segment (residues 49-65) at the C-terminal part of the bound hirudin. Interestingly, the RMSD plot that is based on the full residues, shows some significant local deviations, in the conformation and/or direction of the side-chain of specific residue. Such deviations are observed for the side chains of residues Val1, Glu8, Gln24, Lys27, Ile29, Ser32, Asp33, Glu35, Gln38 and Glu43 (**Figure S18**). In the case of Val1, the different conformation of the residue causes a different positioning of its N-terminal amino moiety, which leads to different interactions with the thrombin active site (*vide supra*). Nevertheless, a close examination of the other

deviating residues shows that their side-chains are not involved in either intra- or intermolecular interactions with the target thrombin,

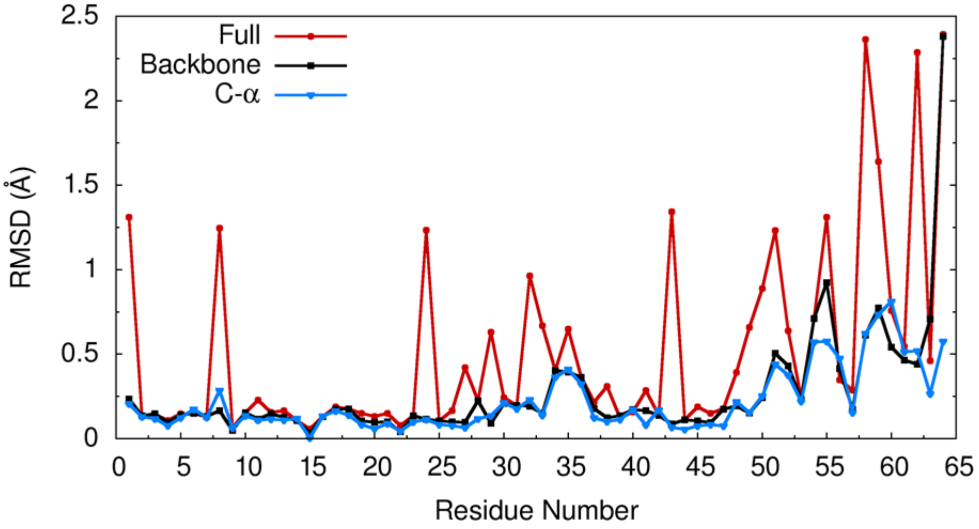

Figure S18. RMSD values between the Hir(C16U/C28U) and Hir(C6U/C14U) in complex with thrombin, respectively, plotted based only on the Cα atoms of the residues (*blue*), only on the backbone atoms (*black*), or based on all atoms of the residue (*red*).

4.4.4.6. Implications of the observed structural differences

The detailed structural analysis of the three Se-Hir derivatives presented here confirmed unequivocally that all three derivatives adopted the same overall conformation as WT-Hir, at least in their bound forms to the thrombin target. Such confirmation is not trivial, considering the replacement of a different disulfide bond with a diselenide bond in each of them, as well as the differences observed in their elution time from HPLC, and their inhibition parameters. The difference was more significant with the Hir(C6U/C14U) analog, compared to the two other Se-Hir analogs and to WT-Hir, emphasizing that this analog behaves differently. In this respect, all four hirudin analogs examined here turned out to be remarkably similar in their overall structure.

Despite the different crystallographic resolutions observed of the complexes, the overall structure and global conformation of all four complexes examined here are nearly identical. Yet, for more reliable comparisons, one should consider primarily the two structures determined in the highest and comparable resolutions, Complex-1 and Complex-2 (determined here at 1.6 Å and 1.9 Å resolution,

respectively). In this respect, Complex-1 could be considered as a general representative of both Complex-3 and the WT-Hir-thrombin complex, all of which showed indeed comparable properties. As such a comprehensive structural comparison was therefore performed on all four complexes, yet focusing mainly on Complex-1 and Complex-2. These analyses included comparisons between intramolecular hydrogen bonds, intermolecular interactions with the target thrombin and local conformational differences per residue. The intermolecular H-bonding between the Se-Hir analogs and thrombin did not identify any significant changes in bonding that are missing or present in any of the structures, except for the one case involving the critical amino-terminus group of Val1. This group, which is likely to be positively charged at neutral pH, makes a slightly different interaction with the active site of thrombin, hydrogen bonding to thrombin Ser205 in Complex-1 while hydrogen bonding to thrombin His43 in Complex-2. In both cases, the hydrogen bond do not seem to be very strong, yet it is probably sufficiently different to lead to the altered binding affinity and inhibition observed for Hir(C16U/C28U), as compared to Hir(C6U/C14U). This change in the conformation of Val1 in Hir(C6U/C14U) is likely a result of the modification of the nearby 6–14 disulfide into a diselenide bond, as further discussed below.

Examination of the intramolecular hydrogen bonds within the Se-Hir structures presented here, demonstrated that they are all quite similar compared to the thrombin-bound form of WT-Hir. Nevertheless, a closer look onto the hirudin globular core showed a tight and conserved network of hydrogen bonds, clustered around residue 6 (Cys/Sec6) in all of the structures compared. Although conserved in all four structures, this clustered network is slightly different in the Hir(C6U/C14U) analog, mainly as a result of the slightly longer 6–14 diselenide bond, which is a disulfide bond in all the other hirudin analogs. Within this tight H-bonding network (**Figure S17**), it seems that the specific hydrogen bonds that Lys47 forms to Asp5 and Thr4, could have a crucial role in positioning the N-terminal peptide of hirudin (residues 1-4) in the active site cleft of thrombin. Moreover, since Asp5 and Thr4 of hirudin are adjacent to the modified residue (Cys/Sec) at position 6, even a slight difference in this segment could lead to altered flexibility, orientation and/or binding capability of this N-terminal peptide. Such a conformational effect may also lead, directly or indirectly, to the slightly different conformation of Val1, located at the free N-terminal end of this peptide.

In retrospect, the tight cluster of intramolecular hydrogen bonds discussed above may account for the robust structure of the hirudin molecule, and the fact that relatively small changes are observed in the

overall structures of the different Se-Hir analogs. Additionally, the location of this cluster close to the hirudin-thrombin interface, and around the 6–14 disulfide/diselenide bond, may explain why the specific replacement of this bond in the Hir(C6U/C14U) analog made more significant changes in its properties, as compared to both Hir(C16U/C28U) and Hir(C22U/C39U), and WT-Hir. Further, the important cluster of intramolecular hydrogen bonds around the 6–14 disulfide further supports its crucial role in the folding mechanism of this protein, explaining, at least in part, the different folding process observed for the Hir(C6U/C14U).

4.5. 2D-NMR of WT-Hir, Hir(C6U/C14U) and Hir(C6U/C16U)

WT-Hir, Hir(C6U/C14U) and Hir(C6U/C16U) samples were all prepared with identical conditions. Proteins were dissolved in 320 µL of 10% D_2O in filtered TDW where the pH was adjusted to 4.5 using solutions of 0.5 M NaOH and 0.1 M HCl. The final ionic strength was approximately 7 mM for all samples. The final concentration of WT-Hir was 1.44 mM, Hir(C6U/C14U) was 1.41 mM and Hir(C6U/C16U) was 0.6 mM.

The experiments were performed under identical conditions on a Bruker AVII 500 MHz spectrometer operating at a proton frequency of 500.13 MHz, using a 5-mm selective probe equipped with a self-shielded XYZ-gradient coil at 18.2 °C. Phase sensitive double quantum filtered correlation spectroscopy (DQF-COSY) experiments[20] were acquired using gradients for water saturation. Spectra were processed, analyzed and presented with TopSpin (Bruker Analytische Messtechnik GmbH) and NMRFAM SPARKY software.[21]

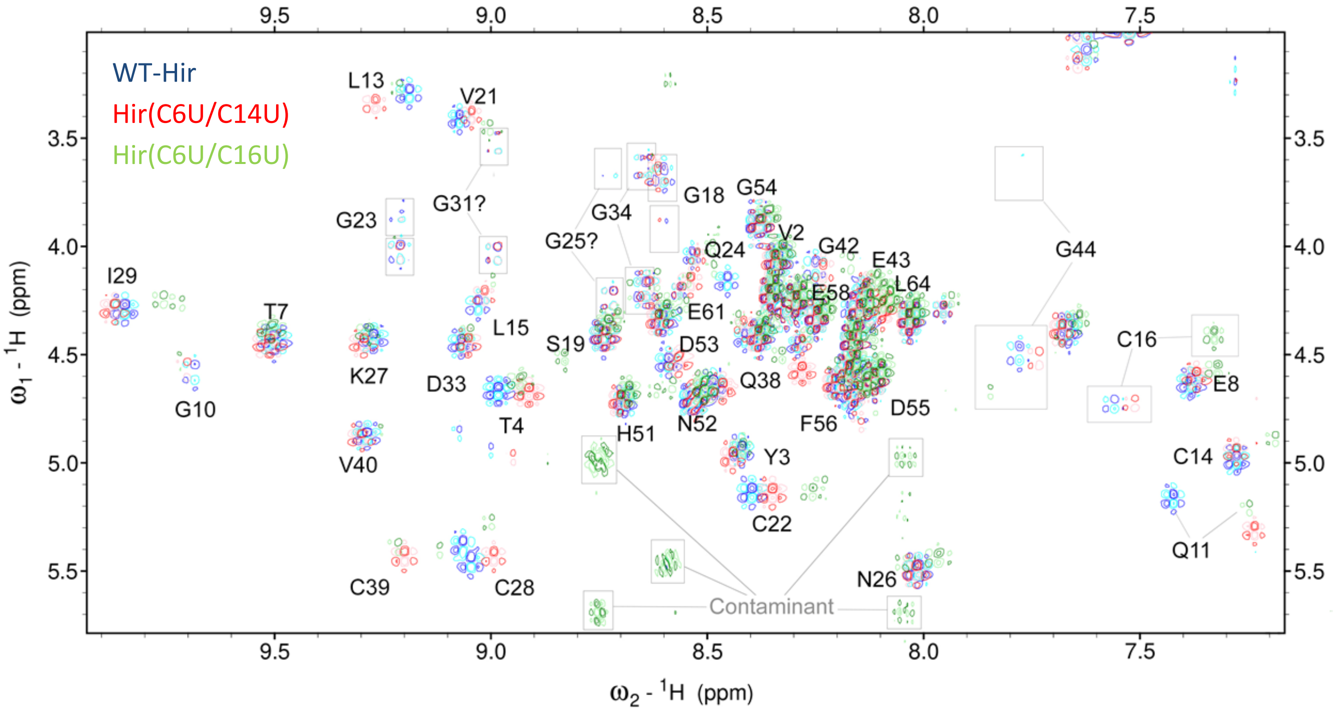

Figure S19. NMR analysis of changes in chemical shift of WT-Hir, Hir(C6U/C14U) and Hir(C6U/C16U). The fingerprint region of NMR COSY spectrum of of Hir(C6U/C14U) (in *red*) and Hir(C6U/C16U) (in *green*) overlaid on that of WT-Hir (in *blue*) showing signals that can be unambiguously assigned according to ref. 22.[22] A contaminant in Hir(C6U/C16U) (*green*), identified by 1D intensities, could not be separated and is noted.

4.6. Inhibition assays

Thrombin activity was assayed in Tris-HCl buffer (50 mM, 154 mM $CaCl_2$, 0.2 % polyethylene glycol 6000, pH 8) at 37 °C.[23] Following pre-incubation of 184 pM of thrombin and inhibitor with a concentration varying between 0-3.7 nM in a total volume of 0.30 mL, the enzymatic reaction was started by the addition of 68.5 µM of N-(*p*-Tosyl)-Gly-Pro-Arg-*p*-nitroanilide (Tos-Gly-Pro-Arg-NH-Np). The initial rate of *p*-nitroaniline formation was followed at 405 nm ($\mathcal{E}_{405}$=9920 $cm^{-1}M^{-1}$) using a Thermo Scientific Evolution™ 201 UV-Visible spectrophotometer. Protein concentration was determined by spectrophotometer ($\mathcal{E}_{280}$ of thrombin is 72150 $cm^{-1}M^{-1}$; $\mathcal{E}_{280}$ of WT-Hir and its seleno-analogs is 2560 $cm^{-1}M^{-1}$). The data were fitted to the following inhibition equation to calculate K_i

$$v = \left(\frac{vo}{2E}\right)\left[\left(\sqrt{(K_i + I - E)^2 + 4K_iE}\right) - (K_i + I - E)\right]$$

The observed K_I values for WT-Hir (10.9 ± 4.9 pM), Hir(C16U/C28U) (10.0 ± 3.7 pM) and Hir(C22U/C39U) (12.5 ± 2.9 pM) are practically identical within experimental error and with excellent agreement with previous literature (10 pM).[23] However the K_I values obtained for Hir(C6U/C14U) (K_I = 192.4 ± 21.9 pM) and Hir(C6U/C16U) (K_I = 104.9 ± 15.0 pM) were higher compared to WT-Hir and its native diselenide containing analogs. All data are shown in Figure 3b and summarized in Table 1 in the main text.

5. HR-MS results

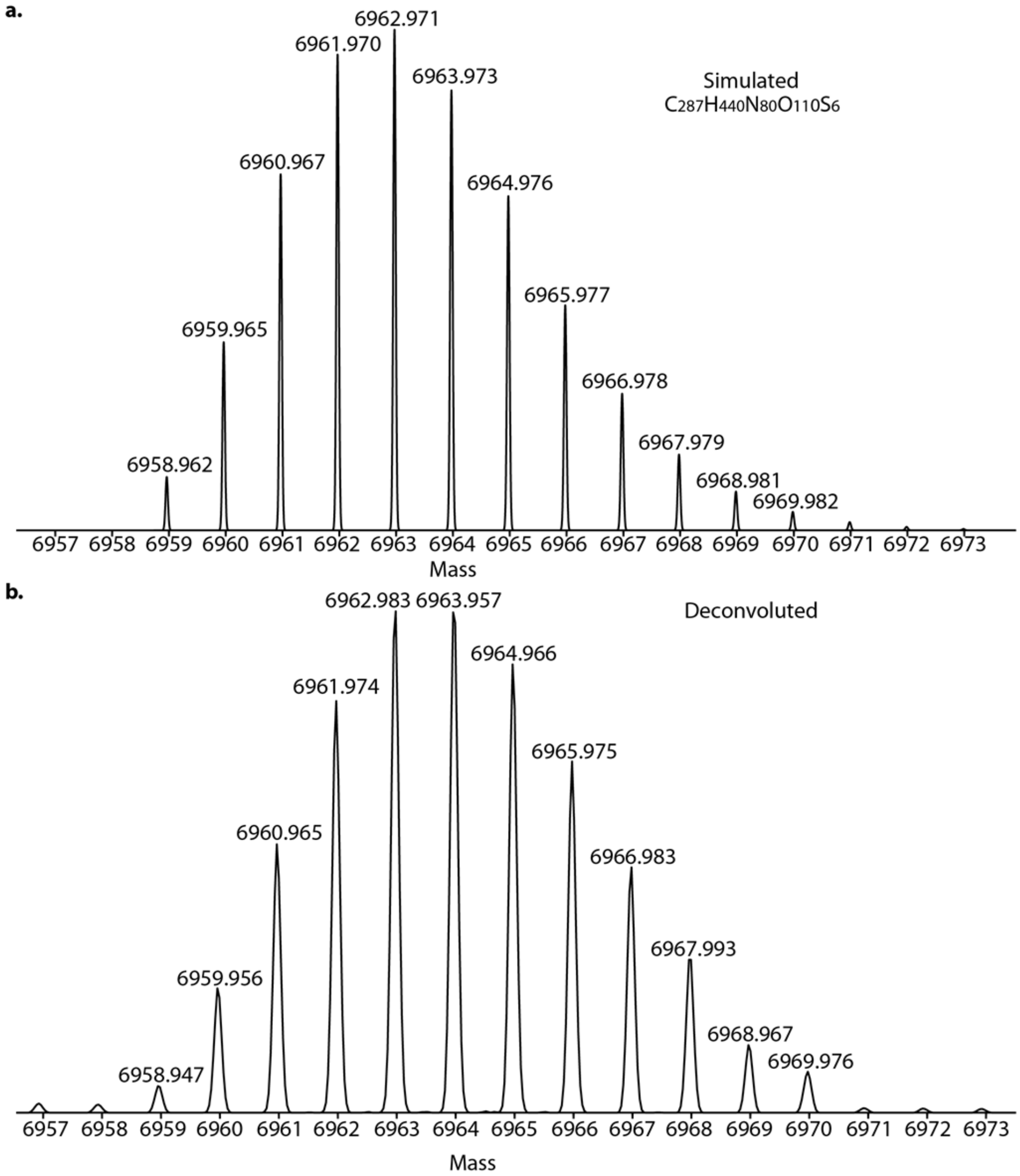

Figure S20. HR-MS analysis of WT-Hir. **a**. The simulated HR-MS of folded WT-Hir with chemical formula $C_{287}H_{440}N_{80}O_{110}S_6$ is shown; **b**. The deconvoluted HR-MS of WT-Hir.

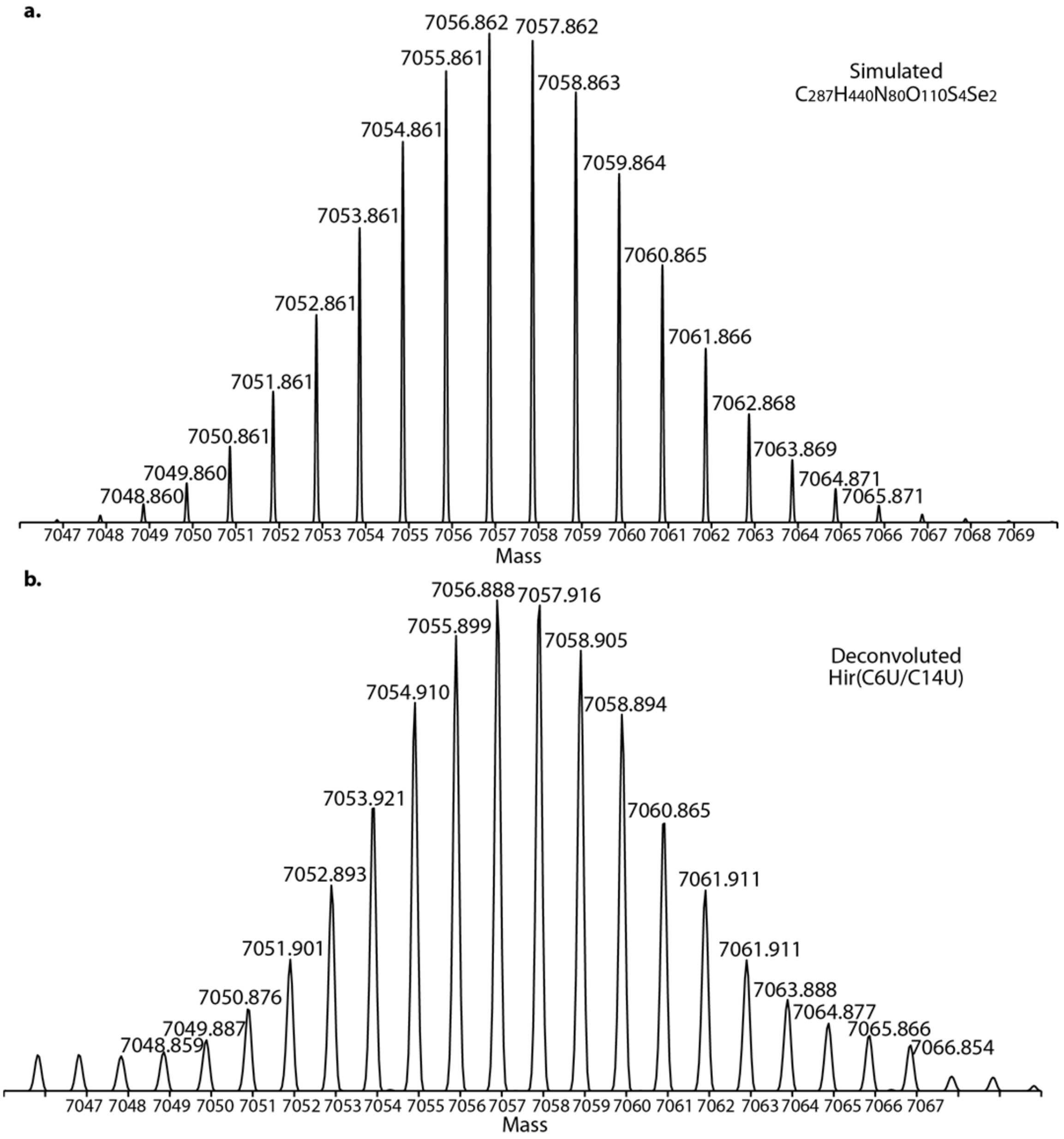
a.
Simulated
C287H440N80O110S4Se2
7056.862
7057.862
7055.861
7058.863
7054.861
7059.864
7053.861
7060.865
7052.861
7061.866
7051.861
7062.868
7050.861
7063.869
7049.860
7064.871
7048.860
7065.871
7047 7048 7049 7050 7051 7052 7053 7054 7055 7056 7057 7058 7059 7060 7061 7062 7063 7064 7065 7066 7067 7068 7069
Mass
b.
Deconvoluted
Hir(C6U/C14U)
7056.888
7057.916
7055.899
7058.905
7054.910
7058.894
7053.921
7060.865
7052.893
7061.911
7051.901
7061.911
7050.876
7063.888
7049.887
7064.877
7048.859
7065.866
7066.854
7047 7048 7049 7050 7051 7052 7053 7054 7055 7056 7057 7058 7059 7060 7061 7062 7063 7064 7065 7066 7067
Mass

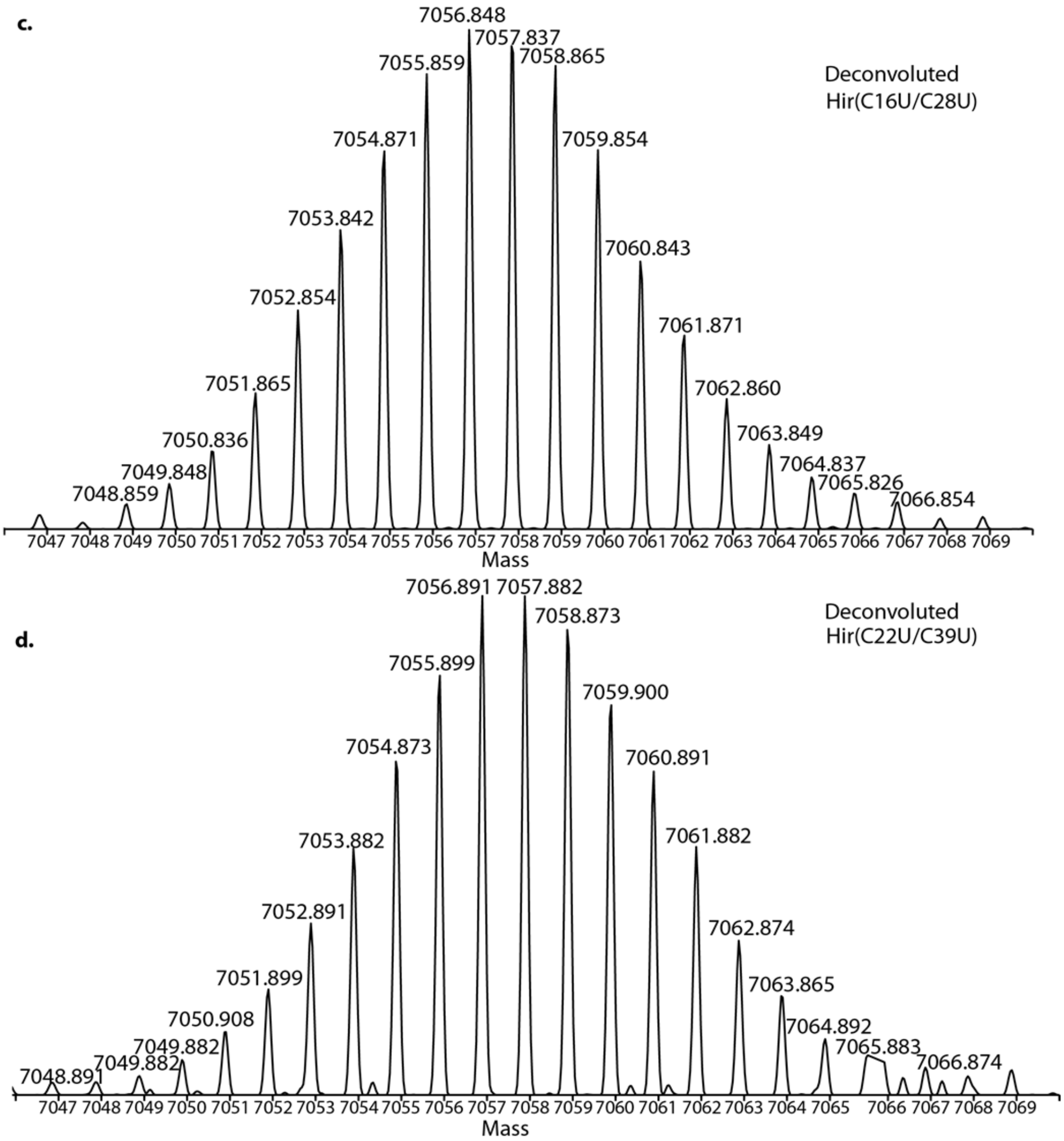
c.
Deconvoluted
Hir(C16U/C28U)
7048.859
7049.848
7050.836
7051.865
7052.854
7053.842
7054.871
7055.859
7056.848
7057.837
7058.865
7059.854
7060.843
7061.871
7062.860
7063.849
7064.837
7065.826
7066.854
7047 7048 7049 7050 7051 7052 7053 7054 7055 7056 7057 7058 7059 7060 7061 7062 7063 7064 7065 7066 7067 7068 7069
Mass
d.
Deconvoluted
Hir(C22U/C39U)
7048.891
7049.882
7049.882
7050.908
7051.899
7052.891
7053.882
7054.873
7055.899
7056.891
7057.882
7058.873
7059.900
7060.891
7061.882
7062.874
7063.865
7064.892
7065.883
7066.874
7047 7048 7049 7050 7051 7052 7053 7054 7055 7056 7057 7058 7059 7060 7061 7062 7063 7064 7065 7066 7067 7068 7069
Mass

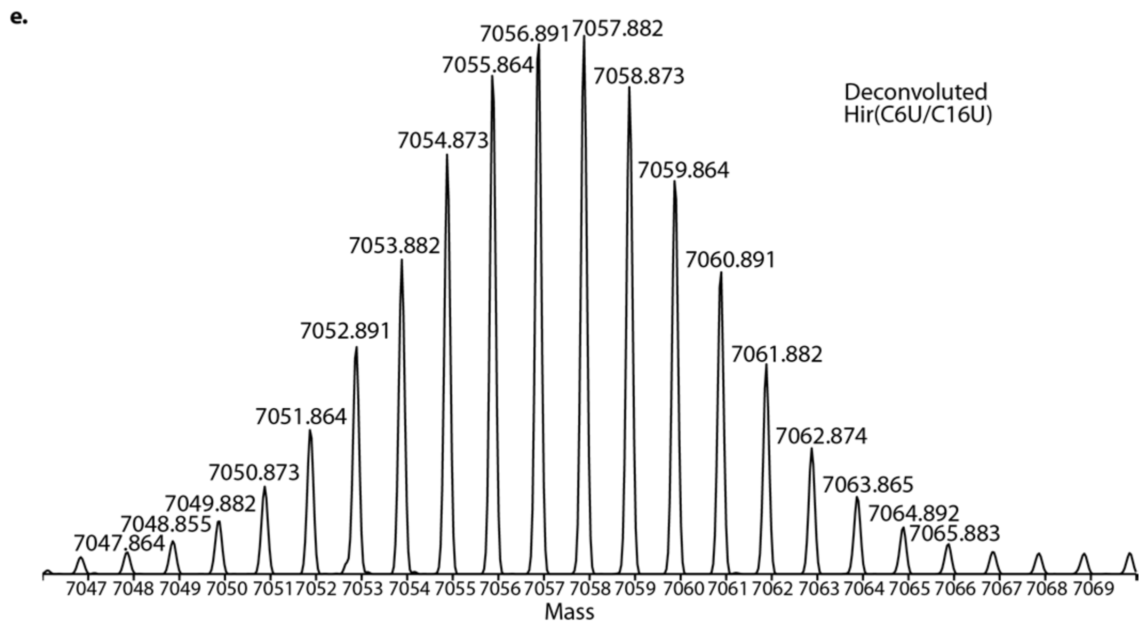

Figure S21. HR-MS analysis of Se-Hir analogs. **a**. The simulated HR-MS of folded diselenide containing hirudin analogs, with the chemical formula $C_{287}H_{440}N_{80}O_{110}S_4Se_2$ shown; The deconvoluted HR-MS of **b**. Hir(C6U/C14U) **c**. Hir(C16U/C28U) **d**. Hir(C22U/C39U) and **e**. Hir(C6U/C16U), are shown as well.

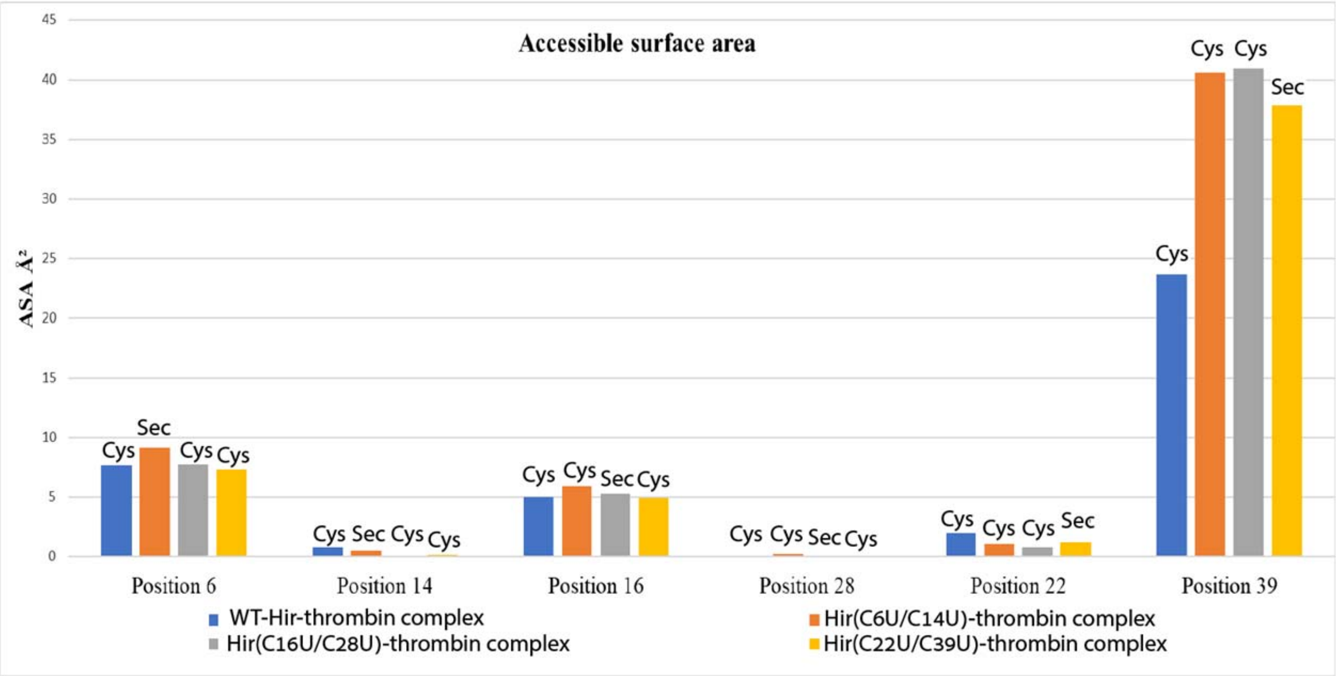

Figure S22. Solvent Accessible Surface Area (SASA) for all Cys and Sec residues in WT-Hir, Hir(C6U/C14U), Hir(C16U/C28U), and Hir(C22U/C39U), complexed with thrombin. The SASA calculations were performed for the Wild-type and the Se-Hirudin thrombin complexes using GETAREA webserver.[24] The calculations showed that both Cys and Sec residues at position 39 are the most exposed compared to positions 6, 14, 16, 22 and 28, of these, the residues at positions 14 and 28 are particularly buried. These calculations are reinforced with the experimental observation of longer HPLC retention time for Hir(C6U/C14U) and Hir(C6U/C16U) analogues. However, there was only a relatively small difference in the ASA between Cys and Sec at the four different positions and when compared to the maximal SASA of cysteine (102.3 Å^2), given at the GETAREA server site, it appears that all the Cys/Sec residues of hirudin are at least somewhat buried.

Supplementray references

[1] P. S. Reddy, S. Dery, N. Metanis, *Angew Chem Int Edit* **2016**, *55*, 992-995.

[2] P. t. Hart, L. H. J. Kleijn, G. de Bruin, S. F. Oppedijk, J. Kemmink, N. I. Martin, *Org. Biomol. Chem.* **2014**, *12*, 913-918.

[3] M. D. Gieselman, L. Xie, W. A. van der Donk, *Org. Lett.* **2001**, *3*, 1331-1334.

[4] J. S. Zheng, S. Tang, Y. K. Qi, Z. P. Wang, L. Liu, *Nat. Protoc.* **2013**, *8*, 2483-2495.

[5] A. L. Schroll, R. J. Hondal, S. Flemer, *J Pept Sci* **2012**, *18*, 1-9.

[6] D. T. Flood, J. C. J. Hintzen, M. J. Bird, P. A. Cistrone, J. S. Chen, P. E. Dawson, *Angew Chem Int Edit* **2018**, *57*, 11634-11639.

[7] J. B. Blanco-Canosa, P. E. Dawson, *Angew. Chem. Int. Ed.* **2008**, *47*, 6851-6855.

[8] B. Chatrenet, J. Y. Chang, *J. Biol. Chem.* **1993**, *268*, 20988-20996.

[9] G. Winter, D. G. Waterman, J. M. Parkhurst, A. S. Brewster, R. J. Gildea, M. Gerstel, L. Fuentes-Montero, M. Vollmar, T. Michels-Clark, I. D. Young, N. K. Sauter, G. Evans, *Acta Crystallogr D* **2018**, *74*, 85-97.

[10] P. R. Evans, G. N. Murshudov, *Acta Crystallographica Section D-Biological Crystallography* **2013**, *69*, 1204-1214.

[11] L. Potterton, J. Agirre, C. Ballard, K. Cowtan, E. Dodson, P. R. Evans, H. T. Jenkins, R. Keegan, E. Krissinel, K. Stevenson, A. Lebedev, S. J. McNicholas, R. A. Nicholls, M. Noble, N. S. Pannu, C. Roth, G. Sheldrick, P. Skubak, J. Turkenburg, V. Uski, F. von Delft, D. Waterman, K. Wilson, M. Winn, M. Wojdyr, *Acta Crystallogr D* **2018**, *74*, 68-84.

[12] A. J. Mccoy, R. W. Grosse-Kunstleve, P. D. Adams, M. D. Winn, L. C. Storoni, R. J. Read, *J Appl Crystallogr* **2007**, *40*, 658-674.

[13] J. Vitali, P. D. Martin, M. G. Malkowski, W. D. Robertson, J. B. Lazar, R. C. Winant, P. H. Johnson, B. F. P. Edwards, *J Biol Chem* **1992**, *267*, 17670-17678.

[14] P. Emsley, B. Lohkamp, W. G. Scott, K. Cowtan, *Acta Crystallographica Section D-Biological Crystallography* **2010**, *66*, 486-501.

[15] O. Kovalevskiy, R. A. Nicholls, F. Long, A. Carlon, G. N. Murshudov, *Acta Crystallogr D* **2018**, *74*, 215-227.

[16] W. Kabsch, *Acta Crystallographica Section D-Biological Crystallography* **2010**, *66*, 125-132.

[17] C. J. Williams, J. J. Headd, N. W. Moriarty, M. G. Prisant, L. L. Videau, L. N. Deis, V. Verma, D. A. Keedy, B. J. Hintze, V. B. Chen, S. Jain, S. M. Lewis, W. B. Arendall, J. Snoeyink, P. D. Adams, S. C. Lovell, J. S. Richardson, D. C. Richardson, *Protein Sci* **2018**, *27*, 293-315.

[18] M. Bauer, H. Brandstetter, D. Turk, J. Sturzebecher, W. Bode, *Semin Thromb Hemost* **1993**, *19*, 352-360.

[19] E. F. Pettersen, T. D. Goddard, C. C. Huang, G. S. Couch, D. M. Greenblatt, E. C. Meng, T. E. Ferrin, *J Comput Chem* **2004**, *25*, 1605-1612.

[20] M. Rance, O. W. Sorensen, G. Bodenhausen, G. Wagner, R. R. Ernst, K. Wuthrich, *Biochem Bioph Res Co* **1983**, *117*, 479-485.

[21] W. Lee, M. Tonelli, J. L. Markley, *Bioinformatics* **2015**, *31*, 1325-1327.

[22] H. Haruyama, K. Wuthrich, *Biochemistry-Us* **1989**, *28*, 4301-4312.

[23] aA. Otto, R. Seckler, *Eur J Biochem* **1991**, *202*, 67-73; bS. R. Stone, J. Hofsteenge, *Biochemistry-Us* **1986**, *25*, 4622-4628.
